# Learner-centered versus teacher-centered dance pedagogy and dance learning and related outcomes: a systematic review

**DOI:** 10.3389/fpsyg.2026.1840900

**Published:** 2026-05-12

**Authors:** Renqing Silang, WeiJia Zhou, Xiaoxue Gao

**Affiliations:** 1 Aba Teachers College, Aba Tibetan and Qiang Autonomous Prefecture, Aba, China; 2 Chengdu Shuangliu Experimental Primary School East District, Chengdu, China; 3 Pukyong National University, Busan, Republic of Korea

**Keywords:** dance education, dance pedagogy, educational psychology, learner-centered teaching, teacher-centered teaching

## Abstract

**Objectives:**

Comparative evidence on learner-centered versus teacher-centered dance pedagogy is fragmented. Thus, the aim of this study was to synthesize the effects of learner-centered and more teacher-directed dance pedagogies on dance learning and related outcomes.

**Methods:**

Eligible studies were original empirical investigations conducted in dance-education settings, including school, studio/conservatory, higher-education, community, extracurricular, and related contexts, using qualitative, mixed-methods, observational, quasi-experimental, or randomized designs. Eligible pedagogical approaches examined learner-centered or more teacher-directed dance teaching, operationalized through features such as learner choice/autonomy, exploration and problem solving, reflection and dialog, collaboration and peer feedback, or direct instruction and demonstration-imitation; eligible outcomes comprised dance learning and performance, motor/physical, motivational-affective, self-related, cognitive, psychosocial, participation-related, and implementation outcomes. Information sources included PubMed, Scopus, and Web of Science. The JBI checklists and RoB 2 were used as risk of bias tools as appropriate.

**Results:**

Among the 38 included original studies, results revealed that learner-centered features (reflection, dialog, collaboration, observation, and choice) were more often associated with more favorable cognitive, self-related, psychosocial, and implementation outcomes. Evidence for superior technical effects over structured teacher guidance was mixed and motor/physical and participation outcomes were sparse. Evidence was heterogeneous and predominantly qualitative and most qualitative studies were moderate quality, while most quasi-experimental studies were high risk of bias.

**Conclusion:**

Overall, the available evidence tentatively suggests that learner-centered elements may enhance several educational outcomes in dance, although conclusions remain tentative because of heterogeneity in study designs, outcomes, and risk of bias. Registration: OSF 2q5v6.

**Systematic review registration:**

Open Science Framework website (Id: 2q5v6, 18/03/2026).

## 1 Introduction

Dance is a complex embodied activity that engages motor experience and expertise, learning and memory, action, intention and emotion understanding, and audio-visual synchrony and timing (Bläsing et al., 2012; Sevdalis and Keller, 2011). Because dance performance and learning depend on the coordination of perceptual, cognitive, motor, and expressive processes, it provides a particularly relevant context for examining how instructional conditions shape skill acquisition and performance (Bläsing et al., 2012; Sevdalis and Keller, 2011). In an embodied domain such as dance, pedagogy is not merely a delivery format since it shapes what learners attend to, how they interpret sensory and verbal information, how they detect and correct error, and how responsibility for movement organization, expression, and adaptation is distributed between teacher and learner (Xuan and Della, 2024). For that reason, differences between more learner-centered and more teacher-directed pedagogy may be especially consequential in dance, where technique, perception, interpretation, and self-regulation are tightly coupled in practice.

Across the wider dance literature, structured dance participation has been associated with a range of psychological and cognitive benefits, and dance is generally as effective as other structured physical activities for several psychological and cognitive health outcomes (Fong Yan et al., 2024). A systematic review focused on aspects of the self concluded that dance interventions can positively influence body-related perceptions, self-trust, self-esteem, self-expression, and perceptions of dance ability, although the evidence base is heterogeneous (Schwender et al., 2018). These outcome domains are closely aligned with the aims of many dance-learning environments, which commonly extend beyond technical execution to include motivation, confidence, agency (learners’ capacity to make meaningful decisions about their movement, learning process, and evaluative adjustment), expression (communication of affect, intention, identity, or artistic meaning through movement choices and performance), and sustained participation (Fong Yan et al., 2024).

Within dance teaching, pedagogical approaches differ in the degree to which learning is organized around exploration, problem solving, and learner decision-making versus direct demonstration, imitation, and reproduction of prescribed movement (Chen, 2001; Guss-West and Wulf, 2016). In this context, learner decision-making refers to learners having meaningful input into aspects of the learning process such as task constraints, pacing and repetition, use of feedback, interpretive choices, peer exchange, and self-assessment (Chen, 2001; Guss-West and Wulf, 2016). Framed this way, the distinction aligns with teaching-style frameworks that differentiate pedagogies partly by how decision-making is distributed between teacher and learner, rather than by a simple opposition between good and bad teaching (Amado et al., 2017; Chen, 2001; Lin et al., 2025).

Some studies indicate that constructivist-oriented creative dance teaching can engage students’ critical thinking, that autonomy-supportive dance teaching can shape motivational processes in school settings, and that creative dance can promote autonomy development in young children (Amado et al., 2017; Chen, 2001; Lin et al., 2025). However, pedagogical processes may differ across dance contexts. In creative and expressive dance, learner-centered approaches may be expected to support divergent movement generation, meaning-making, interpretation, and voice; in technique- and performance-oriented contexts, more structured guidance may remain important for precision, consistency, safety, and style-specific execution (Amado et al., 2017; Chen, 2001; Lin et al., 2025). Instructional variables such as attentional focus, wording of feedback, and broader curricular adaptation have likewise been identified as practically important for dance training and teaching (Guss-West and Wulf, 2016; Wiese et al., 2024; Xuan and Della, 2024). Examples likely to matter include prior dance experience, expertise level, habitual attentional-focus tendencies, and motivational or self-efficacy profiles.

At present, however, the review literature is fragmented across broad benefit-oriented syntheses, self-related outcome reviews, individual-differences frameworks, and narrowly focused instruction-related reviews rather than organized around an explicit comparison between learner-centered and teacher-centered dance pedagogy (Fong Yan et al., 2024; Schwender et al., 2018; Xuan and Della, 2024). As a result, prior studies have not clearly answered the comparative pedagogical question of which instructional features, under which conditions, are associated with more favorable dance-learning and related outcomes (Wiese et al., 2024; Xuan and Della, 2024). They have also left important gaps across populations and settings, particularly in distinguishing school and physical-education contexts from conservatory, studio, and higher-education dance training, and in relating technical/performance outcomes to broader cognitive, self-related, psychosocial, and implementation outcomes within the same synthesis. This fragmentation makes it difficult to determine which pedagogical structures are most consistently associated with favorable dance-learning and related outcomes across contexts, populations, and dance forms (Fong Yan et al., 2024; Schwender et al., 2018; Xuan and Della, 2024). The present review addresses this gap by organizing the literature around pedagogical orientation and instructional features, while also separating direct dance-learning outcomes from broader related outcomes and considering comparative evidence, non-comparative evidence, and implementation evidence in distinct inferential roles. In such scenario, learning and development are understood to include not only technical improvement and dance-task performance, but also cognitive understanding, self-regulation, psychosocial development, expressive capacity, and sustained participation where these outcomes are part of the educational aims of the context.

Accordingly, this systematic review aimed to synthesize the evidence on learner-centered and more teacher-directed dance pedagogies and their reported associations with dance learning and related outcomes. In doing so, it considered not only direct comparative studies, but also broader empirical evidence examining learner-centered pedagogical approaches in relation to usual practice, alternative or more structured instructional formats, and relevant qualitative accounts of pedagogical implementation and learner experience. The review addressed outcomes related to dance performance and learning, as well as motor, motivational, self-related, cognitive, psychosocial, creativity-related, and participation-related outcomes where reported. For interpretive purposes, direct indicators of dance learning and performance were treated as the primary outcome domain, because they are most central to the comparative pedagogical question. The remaining domains were treated as secondary outcome families and were interpreted with attention to evidential density and comparability so that more frequently studied domains would not automatically be given greater inferential weight.

By organizing the literature around pedagogical orientation and instructional features rather than dance participation alone, this review sought to generate evidence that was both conceptually inclusive and instructionally actionable for educators, researchers, and curriculum designers. More specifically, the synthesis was intended to inform decision-relevant guidance at the level of pedagogical components, sequencing of teacher guidance and learner exploration, and implementation conditions such as scaffolding, assessment alignment, and pedagogical fidelity.

## 2 Methods

This systematic review was conducted and reported in accordance with the Preferred Reporting Items for Systematic Reviews and Meta-Analyses (PRISMA 2020). The review question was: How are learner-centered and more teacher-directed dance pedagogies associated with dance learning and related outcomes, and what comparative, experiential, and implementation evidence is available across dance-education contexts?

The systematic review protocol had been established and registered prior to the beginning of the searches. The registration was completed on the Open Science Framework website (2q5v6, 18/03/2026).

### 2.1 Eligibility criteria

Studies were eligible if they were original empirical investigations examining the use, implementation, comparison, or effects of dance pedagogical approaches that could be characterized as learner-centered, student-centered, autonomy-supportive, collaborative, exploratory, improvisational, constructivist, guided-discovery, reciprocal, problem-solving, reflective, or otherwise less instructor-directed. To avoid an unduly narrow evidence base, eligibility was not restricted to studies that explicitly compared a learner-centered condition with a teacher-centered condition. Studies were also eligible where a learner-centered dance pedagogy was examined against usual practice, standard instruction, a more structured or prescriptive format, another pedagogical approach, or in a single-group design, provided that the pedagogical approach was described in sufficient detail and that relevant outcomes were reported. Studies focusing primarily on teacher-centered, command-style, demonstration-imitation, reproduction-based, highly prescriptive, or low-decision-making dance instruction were likewise eligible where these approaches were empirically examined in relation to dance learning or related outcomes. Comparative quantitative studies were treated as the principal basis for any claims about relative effectiveness, whereas qualitative, mixed-methods, and non-comparative studies were used primarily to inform interpretation of mechanisms, learner experience, contextual variation, and implementation. Such evidence was therefore not used on its own to support claims of superiority.

For the purposes of this review, learner-centered pedagogy was defined as instructional approaches that provide meaningful opportunities for exploration, problem solving, improvisation, decision-making, reciprocal learning, peer feedback, guided discovery, divergent production, reflection, self-evaluation, co-construction of learning, or autonomy support within dance learning (Amado et al., 2017; Leisterer and Paschold, 2022). Learner-centered pedagogy was defined multidimensionally rather than as a single technique family. Specifically, eligible approaches gave learners meaningful participation in one or more of three conceptual dimensions: (i) autonomy and choice in how tasks were approached, adapted, or evaluated; (ii) dialogic and reflective processes, including questioning, peer exchange, feedback use, and self-assessment; and (ii) exploratory or generative task design, including problem solving, improvisation, interpretation, or divergent movement production. Teacher-centered pedagogy was defined as instructional approaches characterized predominantly by direct instruction, command or practice style, demonstration-imitation, reproduction of fixed choreography, highly prescriptive task organization, correction-led delivery, or limited learner decision-making (Cothran et al., 2005; Hodges and Franks, 2002). These pedagogical orientations were treated as positions on a continuum rather than as mutually exclusive categories. More specifically, the continuum was treated as multidimensional, with studies positioned according to the distribution of decision rights between teacher and learner, the locus and timing of feedback control, and the openness of the task with respect to reproduction versus interpretation or generation. Instructional episodes were therefore classified by their dominant pedagogical profile across these dimensions rather than by a strict binary label. These categories were used analytically rather than normatively. Direct instruction, demonstration, and tightly structured guidance may be pedagogically appropriate, particularly for novice learners, safety-relevant content, or high-precision technique work, and may be highly effective when paired with opportunities for interpretation, self-monitoring, and gradual release of responsibility.

Studies were eligible regardless of participant age, provided that participants were dance learners, students, dancers, or dance-class participants engaged in educational, recreational, studio-based, conservatory, community, school, extracurricular, informal, or higher-education dance contexts. Studies conducted in physical education contexts were eligible where dance constituted a substantive instructional component rather than a minor or incidental activity. Clinical dance movement therapy, psychotherapy, and rehabilitation studies were excluded unless the intervention was explicitly framed and implemented as dance pedagogy or dance teaching rather than therapy.

Interventions could involve any dance genre or pedagogical form, including but not limited to creative dance, ballet, contemporary dance, modern dance, folk or traditional dance, social dance, DanceSport, jazz, hip-hop, improvisation-based dance, and choreographed educational dance. Studies of creative movement were also eligible where the activity was clearly situated within dance education or dance teaching.

Eligible outcomes included direct indicators of dance learning or performance, such as technique, movement quality, timing, rhythmical or spatial accuracy, coordination, choreography recall, retention, transfer, creativity in movement production, expert or teacher ratings, rubric-based assessments, observational performance measures, or other indicators of dance-task performance. Studies reporting related outcomes were also eligible, including motivation, engagement, enjoyment, autonomy, self-efficacy, self-regulation, confidence, self-related constructs, curiosity, critical thinking, creativity, cognition, psychosocial outcomes, participation, persistence, motor competence, and perceptions or experiences of learning in dance.

A broad range of empirical study designs were eligible, including randomized controlled trials, cluster-randomized trials, non-randomized controlled studies, quasi-experimental studies, crossover studies, repeated-measures comparative designs, pre-post intervention studies, observational comparative studies, longitudinal studies, mixed-methods studies, and qualitative studies that examined the processes, experiences, or perceived effects of learner-centered or teacher-centered dance pedagogy. Qualitative studies were included where they contributed to understanding pedagogical processes, learner experiences, implementation, or mechanisms of effect. Case reports of single individuals, conference abstracts without sufficient data, editorials, commentaries, opinion pieces, and review articles were excluded, although the reference lists of relevant reviews were screened for additional eligible primary studies.

### 2.2 Information sources

Electronic searches were conducted on 18 March 2026 in PubMed, Scopus, and Web of Science Core Collection. No publication-date limits were applied. The reference lists of all included studies and of closely related reviews were also screened manually, and forward citation tracking was undertaken through Scopus and Web of Science for all included studies and selected sentinel papers. Records in any language were considered at the search stage, but studies were included only if sufficient information was available for reliable screening, extraction, and appraisal.

### 2.3 Search strategy

The search strategy was developed iteratively from the review question and refined using terminology identified in related studies and reviews relevant to dance teaching, motor-learning-related instruction, and dance education, including work on constructivist-oriented creative dance teaching, attentional focus and instructional wording, autonomy-supportive dance instruction, autonomy development through creative dance, and pedagogical or curricular adaptation in dance education (Amado et al., 2017; Chen, 2001; Guss-West and Wulf, 2016). The strategy was designed to capture three main concepts: dance, pedagogy and instructional approach.

The following strategy was implemented:

[Title/Abstract/Keywords] dance OR dancing OR dancer* OR ballet OR choreograph* OR “creative dance” OR “dance education” OR “dance technique”

AND

[Title/Abstract/Keywords] pedagog* OR teach* OR instruction* OR curricul* OR “teaching method*” OR “instructional method*” OR “pedagogical approach*” OR “teaching style*” OR “dance teaching” OR “dance training”

AND

[Title/Abstract/Keywords] “student-cent*” OR “learner-cent*” OR “student-led” OR “learner-direct*” OR “guided discover*” OR “divergent discover*” OR “divergent production” OR explor* OR improvis* OR reciprocal OR constructiv* OR collaborative OR “peer feedback” OR “peer assess*” OR reflect* OR “self-evaluat*” OR “autonomy-support*” OR “teacher-cent*” OR “teacher-direct*” OR “teacher-led” OR “direct instruction” OR “command style” OR “practice style” OR “demonstration imitation” OR prescriptive OR reproduction OR “spectrum of teaching styles” OR Mosston

### 2.4 Selection process

All records retrieved from the database and supplementary searches were exported to a Zotero program and deduplicated before screening. Title and abstract screening was performed independently by two authors against the predefined eligibility criteria. Records judged potentially eligible by either author proceeded to full-text assessment. Full texts were then evaluated independently by the same two authors. Reasons for exclusion at the full-text stage were recorded in sufficient detail to permit transparent reporting in the PRISMA flow diagram. Disagreements at any stage were resolved by discussion and, where necessary, by consultation with a third author. Inter-author agreement was quantified at both the title/abstract and full-text stages, showing agreement with *k* = 0.92.

### 2.5 Data collection process

A standardized data extraction form was developed *a priori*, piloted on a small sample of included studies, and refined before full extraction. Two authors extracted data independently from each included study, with discrepancies resolved by consensus or third-author adjudication. When reports were incomplete, ambiguous, or were missing key numerical data, study authors were contacted by email. If numerical outcome data were presented only graphically, values were estimated using Web Plot Digitizer extraction software.

### 2.6 Data items

The primary outcome domain was dance learning, defined as any direct indicator of learning or performance in dance tasks. This included technique execution, movement quality, timing, rhythmical or spatial accuracy, choreography recall, retention, transfer, expert or teacher ratings, rubric-based assessments, and other direct indicators of dance-task performance. Secondary outcome domains included motor and physical outcomes, motivational-affective outcomes, self-related outcomes, cognitive or psychosocial outcomes, participation-related outcomes, and implementation outcomes. Where reported, examples included coordination, balance, enjoyment, intrinsic motivation, autonomy, self-efficacy, self-esteem, self-expression, creativity, curiosity, executive functions, social outcomes, attendance, and adherence.

Additional data items included authors, year, country, study design, setting, participant characteristics, sample size, age or educational stage where available, dance genre, prior dance experience, intervention duration and frequency, total dose, comparator characteristics, teacher qualifications, outcome instruments, assessment time points, and statistical methods.

### 2.7 Study risk of bias assessment

Risk of bias was assessed independently by two authors. As the evidence base was expected to consist mainly of non-randomized and quasi-experimental studies, these studies were appraised using the appropriate Joanna Briggs Institute (JBI) critical appraisal checklist according to design (for example, quasi-experimental, cohort, or cross-sectional studies). Overall judgments were based on the pattern of item-level responses, with particular attention to design-critical domains rather than simple numerical totals alone. For quasi-experimental studies, lack of a control group, major comparability problems, or substantial weaknesses in repeated measurement and follow-up were treated as key threats that could place a study at high risk of bias. For qualitative studies, overall appraisal reflected the coherence of methodology and analysis together with reflexivity, representation of participant voice, and ethics reporting.

Randomized controlled trials were appraised using the revised Cochrane Risk of Bias tool for randomized trials (RoB 2). Disagreements were resolved through discussion and, if necessary, consultation with a third author. Before formal screening, the authors undertook a calibration exercise on an initial sample of records to refine interpretation of the eligibility criteria.

### 2.8 Synthesis methods

Due to anticipated clinical and methodological heterogeneity among the included studies, quantitative synthesis by meta-analysis was not undertaken. Instead, a narrative synthesis was conducted. Included studies were described in evidence tables summarizing study design, participant characteristics, dance form or intervention, comparator (if applicable), outcome measures, follow-up, and principal results.

Narrative synthesis was structured around key review dimensions, including type of dance, characteristics of the study population, study design, and outcome domain. Reported results were presented descriptively, including relevant numerical findings such as means, standard deviations, proportions, change scores, confidence intervals, and *p*-values, where available. The synthesis focused on identifying patterns in the direction, consistency, and apparent magnitude of findings across studies rather than on statistical pooling.

For comparative quantitative outcomes, direction was reported descriptively as favoring more learner-centered pedagogy, favoring more teacher-directed pedagogy, mixed/no clear difference, or unclear. Consistency referred to the extent to which findings converged within a given outcome family across comparable studies and contexts. Apparent magnitude was characterized from reported effect estimates, group differences, change scores, or authors’ results statements where standardized effect sizes were unavailable, and was not treated as equivalent to formal effect-size synthesis. Methodological quality and risk of bias were incorporated by giving greatest interpretive weight to comparative studies with stronger designs and fewer major threats to validity; lower-quality or non-comparative studies were used mainly to elaborate mechanisms, implementation, and contextual interpretation.

Variation in findings was explored in relation to differences in intervention characteristics, duration or frequency, population subgroups, and methodological quality. Risk-of-bias assessments were considered in the interpretation of results, and greater weight was given to studies with more robust methods. The overall conclusions were therefore based on a structured qualitative integration of the available evidence.

## 3 Results

### 3.1 Study selection

**Figure 1:**
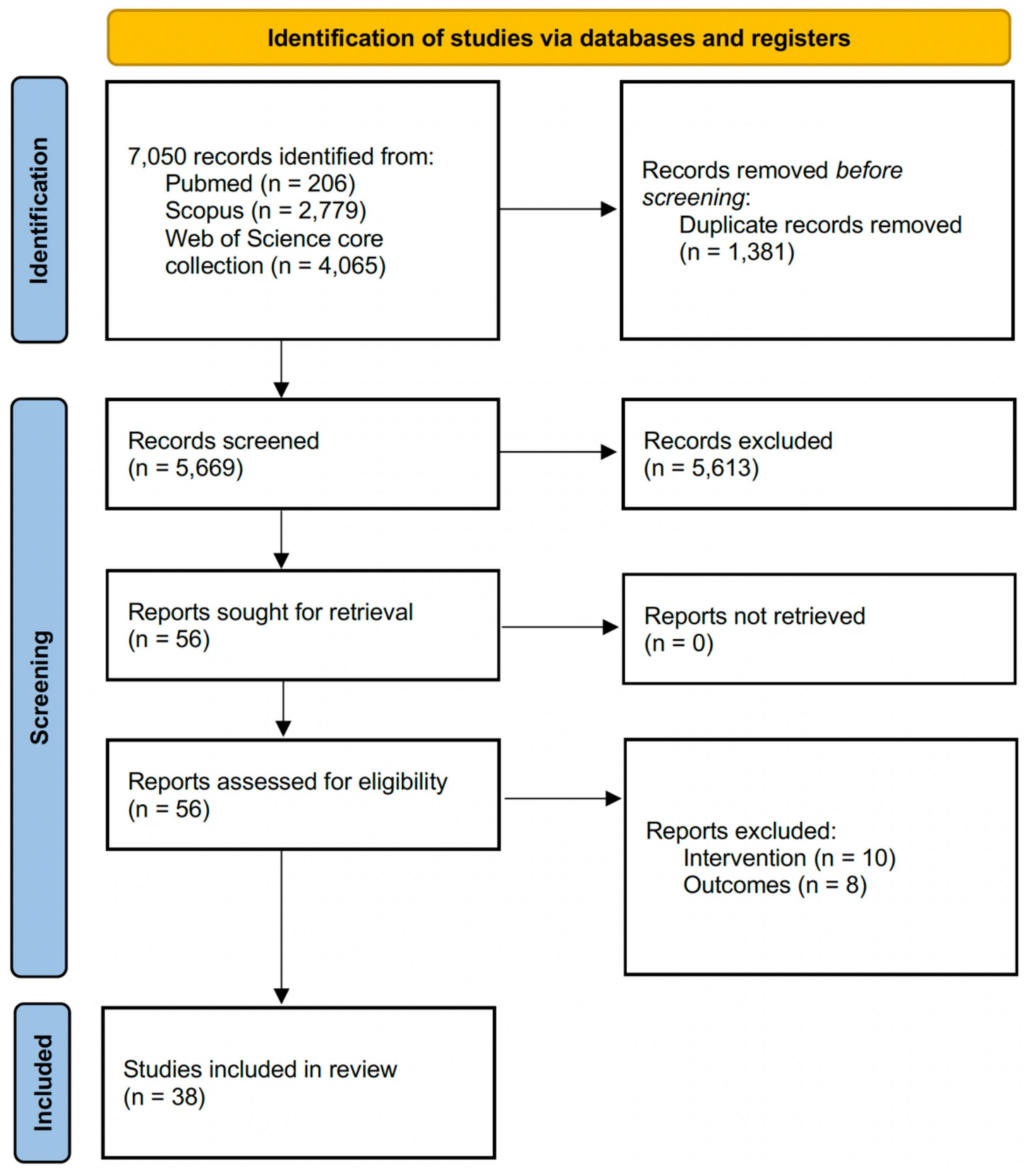
PRISMA flow diagram.

The search identified 7,050 records across PubMed (*n* = 206), Scopus (*n* = 2,779), and Web of Science (*n* = 4,065). Automatic deduplication removed 1,381 duplicate records, leaving 5,669 unique records for screening. Title and abstract screening retained 56 records for full-text review and excluded 5,613 at this stage. Among the 56 full-text reports assessed for eligibility, 17 were excluded. The most frequent reasons for full-text exclusion were ineligible contexts or non-dance-specific pedagogical content (*n* = 10), absence of eligible outcomes (*n* = 8). Accordingly, 38 studies were included in the final synthesis presented in this review (Figure 1).

### 3.2 Study characteristics

The 38 included studies were published between 2009 and 2026. Dance forms and educational contexts were heterogeneous and included ballet, contemporary and modern dance, creative dance, physical education dance, Korean dance, Chinese classical and folk forms, body percussion, critical and feminist dance pedagogy, and broader tertiary dance-education contexts (Table 1). Coding was based on the dominant pedagogical profile of each exposure arm across three dimensions, namely, distribution of decision-making, control and timing of feedback, and task openness with respect to reproduction versus exploration/interpretation. Arms were coded as learner-centered when learner choice, dialogic reflection, peer exchange, exploratory task design, or self−/peer-evaluation were central features; teacher-directed when instruction was predominantly demonstration-led, correction-led, and reproduction-oriented with limited learner decision-making; mixed when substantial teacher structuring and substantial learner-participation features were both present in the same pedagogical design; and unclear when the report did not describe instructional procedures in enough detail for confident placement.

**Table 1:** Characteristics of included studies.

Study	Context	Education stage	Participants	Design	Dance form	Pedagogical focus
Jae-im (2013)	School	Elementary school	Learners, *n* = 17; Teachers or instructors, *n* = 1; Total *n* = 18	Case study	Creative dance and creative movement	Naturalistic creative dance teaching with warm-up, free movement exploration, improvisational and technical skill development, discussion, creating and sharing, collaborative work, and self and group reflection
Andersson (2018)	Upper secondary school	Teacher participants	Teachers or instructors, *n* = 4; Total *n* = 4	Qualitative study	Dance education in upper secondary arts programs; specific genre not limited to one style	Naturalistic assessment and teaching practices in dance education, including teacher demonstration, communicated feedback, student independence in performance, comparison of performances, and varied modes of embodiment
Berg (2017)	Higher education institution	Higher education	Learners, *n* = 42; Teachers or instructors, *n* = 1; Total *n* = 43	Case study	Ballet	IMAGE TECH for Dancers (ITD) integrated Alexander Technique, Irene Dowd’s neuromuscular retraining/ideokinesis, tactile cueing, imagery, anatomical dialog, and self-correction into ballet pedagogy
Brown (2023)	School	Upper secondary school	Learners, *n* = 32; Teachers or instructors, *n* = 1; Total *n* = 33	Mixed-methods study	Ballet and modern dance technique	Student-centered technique pedagogy incorporating peer feedback, student-led teaching, video self-review with student-created rubrics, and student-run discussion/Socratic seminar
Chen (2025)	Extracurricular setting	Mixed ages or stages	Learners, *n* = 215; Teachers or instructors, *n* = 10; Total *n* = 225	Quasi-experimental study	Chinese dance, including Shen Yun and Ribbon Dance	Technology-enhanced dance education program using Presentation Creator, Flipgrid, Dancy, Video Traces, and Dance Designer to support visualization, targeted teacher feedback, rehearsal, and independent study
Choi and Kim (2015)	Mixed settings	Mixed ages or stages	Learners, *n* = 6; Teachers or instructors, *n* = 9; Total *n* = 15	Qualitative study	Ballet	Whole-ballet pedagogy framing ballet education across physical, cognitive, emotional, and spiritual dimensions, with ten direct and indirect teaching methods identified
Cuellar-Moreno and Caballero-Juliá (2019)	Higher education institution	Higher education	Learners, *n* = 50; Teachers or instructors, *n* = 1; Total *n* = 51	Mixed-methods study	Educational dance/Body expression and dance choreography	Comparison of Mosston and Ashworth Command Teaching Style versus Problem Solving Teaching Style in dance classes
Dryburgh (2019)	Higher education institution	Higher education	Learners, *n* = 7; Teachers or instructors, *n* = 1; Total *n* = 8	Longitudinal observational study	Contemporary dance/release-based contemporary dance technique	Engaged-pedagogy and care-oriented contemporary dance technique teaching emphasizing active participation, student filtering and application of information, reflection on bodily experience, peer stimulation, and teacher responsiveness
Engdahl et al. (2024)	Higher education institution	Higher education	Learners, *n* = 11; Teachers or instructors, *n* = 1; Total *n* = 12	Qualitative study	Creative dance in physical education teacher education	Creative dance pedagogy based on imitation, mirroring, collaborative planning, discussion, and reflection to open spaces of experimentation and unpredictable movement exploration
Feltham and Ryan (2022)	Mixed settings	Teacher participants	Teachers or instructors, *n* = 4; Total *n* = 4	Qualitative study	Ballet	Interview-based characterization of pedagogical and recruitment practices for engaging boys in ballet and for including gender non-conforming children, including male role models, media use, same-technique mixed-class teaching, inclusive language, and costume/movement choice
García-Dantas and Quested (2015)	Dance studio or conservatory	Mixed ages or stages	Learners, *n* = 61; Teachers or instructors, *n* = 5; Total *n* = 66	Randomized controlled trial	Flamenco and Spanish dance; Zapateado footwork task	Comparison of upper-evaluated feedback, objective and informational accurate feedback, lower-evaluated feedback, and no-feedback control during repeated execution of a Zapateado sequence
Guo and Thiagarajan (2025)	Higher education institution	Higher education	Learners, *n* = 16; Teachers or instructors, *n* = 1; Total *n* = 17	Qualitative study	ChaoXian folk dance	Laban/Bartenieff Movement System pedagogy integrating the Body, Effort, Space, and Shape framework, somatic methods, experiential learning, discussion, summarization, and free exploration in ChaoXian dance education
Hsia (2020)	Higher education institution	Higher education	Learners, *n* = 65; Teachers or instructors, *n* = 1; Total *n* = 66	Mixed-methods study	General education dance course and group dance performance task	Flipped learning using teacher-recorded instructional videos plus anonymous mobile technology-supported peer assessment during group performance
Huddy (2017)	Higher education institution	Higher education	Not reported	Mixed-methods study	Contemporary dance, ballet, jazz, Latin, and hip hop technique	Digitally enhanced progressive assessment using mobile video capture, digital repositories, inquiry, reflective analysis, and oral presentation to build student agency, collaboration, and responsibility for learning
Jin-hee (2010)	Higher education institution	Higher education	Learners, *n* = 12; Teachers or instructors, *n* = 1; Total *n* = 13	Qualitative study	Modern dance/contemporary dance creation	Portfolio assessment integrated self-evaluation, peer evaluation, group evaluation, reflective journaling, staged creative tasks, and media-supported review into a contemporary dance creation class
Jo and Lee (2022)	Physical education context	Teacher participants	Teachers or instructors, *n* = 10; Total *n* = 10	Qualitative study	Folk dance as expressive activity in school physical education	Teacher participants described group-centered, interest-centered, theme-centered, and skill-centered folk dance teaching-learning content, while also emphasizing the need for stronger systems, teacher expertise, and clearer evaluation standards
Eun-ju and Dae-jung (2020)	Higher education institution	Higher education	Teachers or instructors, *n* = 1; Total *n* = 1	Qualitative study	Liberal arts dance classes including jazz dance, folk dance, and sport dance	Teacher self-study traced a shift from high-pressure, teacher-centered teaching toward a learner-responsive, relational pedagogy emphasizing student understanding, shared value, dance emotion, and growth guidance
Jung et al. (2026)	Mixed settings	Mixed ages or stages	Learners, *n* = 6; Teachers or instructors, *n* = 12; Total *n* = 18	Qualitative study	Korean dance	Practice-based Korean dance pedagogy organized as Transmission, Facilitation, and Co-construction
Park (2017)	Mixed settings	Adult community learners	Teachers or instructors, *n* = 5; Total *n* = 5	Qualitative study	Elderly dance education across contemporary dance, Korean dance, and ballet-informed practice	Esthetic-development-based pedagogy for elderly dance education emphasizing body, movement, emotion, and expression
Larsson et al. (2025)	Higher education institution	Higher education	Learners, *n* = 50; Teachers or instructors, *n* = 2; Total *n* = 52	Qualitative study	Creative dance/expressive dance	Teacher-guided creative-dance pedagogy using movement-phrase exploration, performance structuring, and the Laban movement analysis framework
Longley and Kensington-Miller (2020)	Higher education institution	Higher education	Learners, *n* = 150; Teachers or instructors, *n* = 2; Total *n* = 152	Mixed-methods study	Dance studies/tertiary dance education	Pedagogy for making invisible graduate attributes explicit through narrative reflection and the SEEN framework
Ma (2024)	Higher education institution	Higher education	Learners, *n* = 130; Teachers or instructors, *n* = 3; Total *n* = 133	Quasi-experimental study	Chinese classical dance	Online dance-analysis pedagogy combining digital resource retrieval, movement recognition, multimedia support, and multicultural resource integration
Mabingo (2019)	Mixed settings	Higher education	Learners, *n* = 6; Total *n* = 6	Qualitative study	Ugandan African neo-traditional dance	Experiential and reflective study-abroad pedagogy using local Ugandan dance and music instruction, storytelling, collaborative lesson-planning, co-teaching, and embodied intercultural participation
Marani et al. (2022)	Higher education institution	Higher education	Not reported	Qualitative study	Dance in physical education; ballroom dance	Physical-Cultural-Studies-informed dance pedagogy emphasizing democratic dialog, critical reflection, partner exchange, innovative leading practices, and critical analysis of public pedagogies about dance
Mattsson and Larsson (2021)	Physical education context	Middle school	Learners, *n* = 68; Teachers or instructors, *n* = 4; Total *n* = 72	Qualitative study	Expressive dance	Teacher-guided expressive-dance pedagogy using exploratory assignments, progressive risk-taking, and Rudolf Laban’s Body, Effort, Space, and Shape framework
McCarthy-Brown (2021)	Mixed settings	Mixed ages or stages	Learners, *n* = 42; Teachers or instructors, *n* = 1; Total *n* = 43	Mixed-methods study	Critical dance pedagogy and choreographic repertory process	Critical dance pedagogy using embodied dialog about race and power, student-generated movement, choreographic creation, reflection, and public performance
Mi-Hee (2016)	Higher education institution	Higher education	Teachers or instructors, *n* = 1; Total *n* = 1	Qualitative study	Dance education for prospective teachers, including folk-dance and expressive movement content	Autoethnographically reflected teaching trajectory from rigid training and practical-skill instruction toward enjoyable, learner-responsive, culturally meaningful dance education for prospective teachers
Jin-kyung et al. (2009)	Dance studio or conservatory	Elementary school	Learners, *n* = 32; Teachers or instructors, *n* = 4; Total *n* = 36	Observational comparative study	Cecchetti ballet	Predominantly teacher-directed Cecchetti ballet instruction examined through complete demonstration, cueing, and task-focused feedback quality
Ørbæk and Engelsrud (2021)	Mixed settings	Higher education	Learners, *n* = 8; Total *n* = 8	Case study	Creative dance in physical education	Creative-dance pedagogy evolving from set-movement instruction toward expressive exploration and movement-emotion-language connections
Park (2017)	School	Mixed ages or stages	Learners, *n* = 15; Total *n* = 15	Qualitative study	Dance-centered integrated arts education	Esthetic-experience-oriented dance education emphasizing imagination, expression, creation, appreciation, varied stimuli, autonomous methods, and group activity; the study derived the Ae-SD strategy
Powell (2025)	Higher education institution	Higher education	Teachers or instructors, *n* = 1; Total *n* = 1	Case study	Cunningham-based contemporary dance technique	Feminist and democratic dance-technique pedagogy using open questions, discussion, shared decision-making, collaboration, and differentiation to challenge authoritarian teaching
Rimmer (2017)	Higher education institution	Higher education	Learners, *n* = 7; Teachers or instructors, *n* = 1; Total *n* = 8	Qualitative study	Integrated contemporary dance technique drawing on Cunningham, Hawkins, and release-based ideas	Enquiry-based, constructivist dance-technique pedagogy using partner problem-solving, phrase creation, improvisation, discussion, and journal reflection to support active learner agency
Rimmer-Piekarczyk (2018)	Higher education institution	Higher education	Learners, *n* = 7; Teachers or instructors, *n* = 1; Total *n* = 8	Qualitative study	Release-based contemporary dance technique	Dialogic, somatic-informed teaching that returned questions to students, foregrounded first-person reflection, used exploratory tasks, peer observation, peer feedback, improvisation, and collaborative discussion to challenge hierarchical technique teaching
Rothmund (2023)	Higher education institution	Higher education	Learners, *n* = 11; Total *n* = 11	Qualitative study	Contemporary dance techniques including Graham, Cunningham, release-based techniques, improvisation, and contact improvisation	The study analyzed student learning experiences across a continuum from teacher-centered reproductive technique work toward more student-centered explorative, improvisational, and self-regulated learning practices
Soerel et al. (2023)	Higher education institution	Higher education	Teachers or instructors, *n* = 6; Total *n* = 6	Observational comparative study	Modern Jazz, improvisation, ballet, Graham, Laban, and Graham repertoire rehearsal	The study mapped naturally occurring instruction, questioning, feedback, and focus-of-attention practices to characterize how teaching information was delivered across class types.
Swain et al. (2025)	Higher education institution	Higher education	Learners, *n* = 62; Teachers or instructors, *n* = 1; Total *n* = 63	Pre-post intervention study	Body percussion within Body Expression and Dance education	Specialized body-percussion pedagogy integrated into Body Expression and Dance, using rhythmic motor games, cooperative participation, rotating artistic roles, and musical improvisation to explore body-time relations
Vincent et al. (2021)	School	Upper secondary school	Teachers or instructors, *n* = 8; Total *n* = 8	Mixed-methods study	Certificated school dance including GCSE and National 5 work in styles such as jazz and contemporary, linked to professional dance works	The study compared teacher-reported delivery of certificated dance curricula, highlighting a more integrated, resource-based and partially student-centered GCSE approach versus more compartmentalized National 5 delivery with variable discovery learning
Wilson (2009)	Higher education institution	Higher education	Learners, *n* = 51; Teachers or instructors, *n* = 1; Total *n* = 52	Mixed-methods study	Contemporary dance training, ballet technique, experiential anatomy, and rond de jambe ballet movement analysis	Somatic experiential-anatomy pedagogy that presented anatomical and kinesiological concepts and then asked dancers to explore, embody, journal, discuss, and apply them in movement and technique contexts

Despite this heterogeneity, the evidence clustered in recurring contexts. Ballet and other technique-focused studies more often examined feedback, correction, somatic awareness, and performance quality; creative-dance and physical-education studies more often emphasized exploration, meaning-making, creativity, and participation; and higher-education contemporary, dialogic, and critical-pedagogy studies contributed much of the evidence on reflection, agency, dialog, and sociocultural learning.

Implementation outcomes (Table 2) were the most frequently reported domain, followed by cognitive outcomes, self-related outcomes, and dance learning and performance, whereas motor and physical outcomes and participation-related outcomes were comparatively rare. Implementation outcomes dominated largely because many studies were qualitative or mixed-methods investigations of enactment rather than controlled tests of performance change. Common implementation themes included feasibility, learner readiness, assessment practices, teacher and student perceptions, and the institutional constraints shaping whether learner-centered pedagogy could be sustained in practice.

**Table 2:** Distribution of the outcome-domain coverage.

Outcome domain	Studies reporting the domain	Outcome records
Implementation outcomes	28	38
Cognitive outcomes	24	26
Self-related outcomes	24	30
Dance learning and performance	21	24
Psychosocial outcomes	17	17
Motivational-affective outcomes	14	21
Motor and physical outcomes	5	5
Participation-related outcomes	4	4

Outcome-domain coverage is reported as the number of studies contributing at least one outcome record and the total number of extracted outcome records in that domain.

### 3.3 Methodological quality and risk of bias

Among the 32 studies appraised with the Joanna Briggs Institute qualitative checklist (Table 3), 29 were judged moderate, 1 low, and 2 high overall. Most qualitative studies clearly aligned methods with the research question and interpretation, and most adequately represented participant voices, but reporting of philosophical positioning, reflexivity, and ethics was less consistent.

**Table 3:** Appraisal matrix for studies assessed with the Joanna Briggs Institute qualitative checklist.

Study	Item 1	Item 2	Item 3	Item 4	Item 5	Item 6	Item 7	Item 8	Item 9	Item 10	Overall appraisal
Jae-im (2013)	Unclear	Yes	Yes	Unclear	Yes	No	No	Yes	No	Yes	Moderate
Andersson (2018)	Yes	Yes	Yes	Yes	Yes	Yes	Unclear	Yes	Yes	Yes	Moderate
Berg (2017)	Unclear	Yes	Yes	Yes	Yes	Yes	Unclear	Yes	Yes	Yes	Moderate
Choi and Kim (2015)	Yes	Yes	Yes	Yes	Yes	No	No	Yes	Yes	Yes	Moderate
Cuellar-Moreno and Caballero-Juliá (2019)	Unclear	Yes	Yes	Yes	Yes	No	No	Yes	Yes	Yes	Moderate
Dryburgh (2019)	Yes	Yes	Yes	Yes	Yes	Yes	Yes	Yes	Yes	Yes	Moderate
Engdahl et al. (2024)	Yes	Yes	Yes	Yes	Yes	Yes	Unclear	Yes	Yes	Yes	Moderate
Feltham and Ryan (2022)	Yes	Yes	Yes	Yes	Yes	No	No	Yes	Yes	Yes	Moderate
Guo and Thiagarajan (2025)	Yes	Yes	Yes	Yes	Yes	Yes	Yes	Yes	Yes	Yes	Low
Huddy (2017)	Unclear	Yes	Yes	Unclear	Yes	Yes	Yes	Yes	Yes	Yes	Moderate
Jin-hee (2010)	Unclear	Yes	Yes	Yes	Yes	Yes	Unclear	Yes	No	Yes	Moderate
Jo and Lee (2022)	Unclear	Yes	Yes	Yes	Yes	No	No	Yes	Yes	Yes	Moderate
Eun-ju and Dae-jung (2020)	Unclear	Yes	Yes	Yes	Yes	Yes	Yes	Yes	No	Yes	Moderate
Jung et al. (2026)	Unclear	Yes	Yes	Yes	Yes	Yes	Unclear	Yes	Yes	Yes	Moderate
Park (2017)	Unclear	Yes	Yes	Yes	Yes	No	No	Yes	Yes	Yes	Moderate
Larsson et al. (2025)	Unclear	Yes	Yes	Yes	Yes	Yes	Unclear	Yes	No	Yes	Moderate
Longley and Kensington-Miller (2020)	Unclear	Yes	Yes	Unclear	Yes	Yes	Unclear	Unclear	Yes	Yes	Moderate
Mabingo (2019)	Unclear	Yes	Unclear	Unclear	Yes	Unclear	Unclear	Unclear	Unclear	Unclear	High
Marani et al. (2022)	Yes	Yes	Yes	Unclear	Yes	Yes	Yes	Unclear	No	Yes	Moderate
Mattsson and Larsson (2021)	Yes	Yes	Yes	Yes	Yes	No	No	Unclear	Yes	Yes	Moderate
McCarthy-Brown (2021)	Yes	Yes	Yes	Yes	Yes	Yes	Yes	Yes	Yes	Yes	Moderate
Mi-Hee (2016)	Unclear	Yes	Yes	Yes	Yes	Yes	Unclear	Yes	No	Yes	Moderate
Jin-kyung et al. (2009)	Unclear	Yes	Yes	No	No	No	Unclear	Yes	Yes	Unclear	Moderate
Ørbæk and Engelsrud (2021)	Yes	Yes	Yes	Yes	Yes	Unclear	Unclear	Yes	No	Yes	Moderate
Park (2017)	Yes	Yes	Yes	Yes	Yes	Unclear	Unclear	Yes	No	Yes	Moderate
Powell (2025)	Yes	Yes	Unclear	Unclear	Yes	Yes	Yes	No	No	Yes	High
Rimmer (2017)	Unclear	Yes	Yes	Yes	Yes	Yes	Yes	Yes	Yes	Yes	Moderate
Rimmer-Piekarczyk (2018)	Unclear	Yes	Yes	Yes	Yes	Yes	Yes	Yes	Yes	Yes	Moderate
Rothmund (2023)	Yes	Yes	Yes	Yes	Yes	Unclear	No	Yes	Yes	Yes	Moderate
Soerel et al. (2023)	Yes	Yes	Yes	Unclear	No	No	Yes	Yes	Unclear	Unclear	Moderate
Vincent et al. (2021)	Yes	Yes	Yes	Yes	Yes	Yes	Unclear	Yes	Yes	Yes	Moderate
Wilson (2009)	Yes	Yes	Yes	Yes	Yes	Yes	Yes	Yes	Yes	Yes	Moderate

Item numbering follows the Joanna Briggs Institute qualitative checklist sequence. Item 1, congruity between philosophical perspective and methodology; Item 2, congruity between methodology and research question or objectives; Item 3, congruity between methodology and data collection; Item 4, congruity between methodology and data representation and analysis; Item 5, congruity between methodology and interpretation; Item 6, researcher located culturally or theoretically; Item 7, influence of the researcher addressed; Item 8, participant voices adequately represented; Item 9, ethics approval or ethical process reported; Item 10, conclusions flow from the analysis.

Of the 5 quasi-experimental studies (Table 4), 4 were judged high and 1 moderate overall. The most recurrent weaknesses were lack of a control group or limited comparability of groups, incomplete pre-intervention and post-intervention measurement structures, and insufficient handling of follow-up and confounding.

**Table 4:** Appraisal matrix for studies assessed with the Joanna Briggs Institute quasi-experimental checklist.

Study	Item 1	Item 2	Item 3	Item 4	Item 5	Item 6	Item 7	Item 8	Item 9	Overall appraisal
Brown (2023)	Yes	Yes	Unclear	Yes	No	Yes	Yes	Yes	Yes	Moderate
Chen (2025)	Yes	Unclear	Unclear	No	No	Unclear	Yes	Unclear	Unclear	High
Cuellar-Moreno and Caballero-Juliá (2019)	Yes	Yes	Yes	No	No	Unclear	Yes	Yes	No	High
Ma (2024)	Yes	Unclear	Unclear	Yes	No	Unclear	Yes	Unclear	Unclear	High
de Garcías Ves and Beltran-Garrido (2024)	Yes	Yes	Yes	No	Yes	No	Yes	Yes	Yes	High

Item numbering follows the Joanna Briggs Institute quasi-experimental checklist sequence: clarity of cause and effect; similarity of participants across comparisons; similarity of care apart from the intervention; presence of a control group; multiple pre-intervention and post-intervention measurements; completeness of follow-up; consistency of outcome measurement; reliability of outcome measurement; and appropriateness of statistical analysis.

Table 5 shows the appraisal matrix for studies assessed with the revised Cochrane Risk of Bias tool for randomized trials.

**Table 5:** Appraisal matrix for studies assessed with the revised Cochrane risk of bias tool for randomized trials.

Study	Randomization process	Deviations from intended interventions	Missing outcome data	Measurement of outcome	Selection of the reported result	Overall appraisal
García-Dantas and Quested (2015)	Some concerns	Low	Low	Some concerns	Some concerns	Some concerns
Hsia (2020)	Some concerns	Low	Low	Some concerns	Some concerns	Some concerns

### 3.4 Characteristics of pedagogical approaches and intervention protocols

Forty-six exposure arms were identified across the 38 studies. Twenty-two arms were coded as learner-centered, 8 as more teacher-directed, and 16 as occupying a mixed or unclear position on the learner-centered to teacher-directed continuum. Exposure most often reflected ongoing routine pedagogy (20 arms) or interventions involving more than one exposure session (19 arms); only 7 arms were acute or single-exposure designs.

The most prevalent technique families were autonomy support and learner choice (22 studies), discussion and verbal sense-making (21 studies), direct instruction and demonstration (19 studies), collaborative learning and peer interaction (18 studies), and reflection and self-evaluation (18 studies). Table 6 details the pedagogical approaches, contextual contrasts, delivery patterns, reported dose, and core technique families for each study.

**Table 6:** Characteristics of pedagogical approaches, contextual contrasts, and intervention protocols.

Study	Pedagogical approach or intervention	Comparator or contextual contrast	Duration	Teacher qualifications	Core technique families	Outcome domains reported
Jae-im (2013)	Naturalistic creative dance teaching with warm-up, free movement exploration, improvisational and technical skill development, discussion, creating and sharing, collaborative work, and self and group reflection	No comparator	5 sessions	Teacher had master’s-level study at New York University and more than 10 years of dance background and teaching experience	Improvisation and movement exploration; Collaborative learning and peer interaction; Reflection and self-evaluation; Direct instruction and demonstration; Discussion and verbal sense-making	Dance learning and performance; Motor and physical outcomes; Cognitive outcomes; Psychosocial outcomes; Self-related outcomes
Andersson (2018)	Naturalistic assessment and teaching practices in dance education, including teacher demonstration, communicated feedback, student independence in performance, comparison of performances, and varied modes of embodiment	No comparator	Not reported	Teachers had varied educational backgrounds, including dance pedagogy education, professional dance education, professional dancer experience, and teaching experience	Direct instruction and demonstration; Autonomy support and learner choice; Observation and performance comparison; Feedback and assessment practices; Reflection and self-evaluation	Implementation outcomes; Dance learning and performance; Motivational-affective outcomes; Psychosocial outcomes
Berg (2017)	IMAGE TECH for Dancers (ITD) integrated Alexander Technique, Irene Dowd’s neuromuscular retraining/ideokinesis, tactile cueing, imagery, anatomical dialog, and self-correction into ballet pedagogy	No comparator	Not reported	Teacher was American-born, trained in the United States, danced professionally, and had a 15-year career as a ballet soloist in France	Feedback and assessment practices; Reflection and self-evaluation; Observation and performance comparison; Discussion and verbal sense-making; Practice and repetition	Dance learning and performance; Motor and physical outcomes; Self-related outcomes; Implementation outcomes
Brown (2023)	Student-centered technique pedagogy incorporating peer feedback, student-led teaching, video self-review with student-created rubrics, and student-run discussion/Socratic seminar	Teacher-centered direct instruction with teacher-led warm-up, centre/across-floor combinations, and teacher-provided feedback	8 sessions; 8 weeks	Classroom teacher-researcher held dance training and graduate education credentials; external adjudicators had dance performance/choreography credentials	Direct instruction and demonstration; Collaborative learning and peer interaction; Composition and choreography creation; Feedback and assessment practices; Discussion and verbal sense-making	Dance learning and performance; Self-related outcomes; Cognitive outcomes
Chen (2025)	Technology-enhanced dance education program using Presentation Creator, Flipgrid, Dancy, Video Traces, and Dance Designer to support visualization, targeted teacher feedback, rehearsal, and independent study	No true comparator; two learner groups studied classical versus contemporary Shen Yun within the same feedback-based program	50 min per session	Ten educators participated, each with at least 8 years of dance teaching experience	Feedback and assessment practices; Practice and repetition; Observation and performance comparison; Autonomy support and learner choice	Dance learning and performance; Motivational-affective outcomes; Implementation outcomes; Participation-related outcomes
Choi and Kim (2015)	Whole-ballet pedagogy framing ballet education across physical, cognitive, emotional, and spiritual dimensions, with ten direct and indirect teaching methods identified	No comparator	Not reported	Teachers were experienced ballet educators from conservatory, school-based, and private-studio sectors	Direct instruction and demonstration; Practice and repetition; Discussion and verbal sense-making; Guided discovery and problem solving; Autonomy support and learner choice; Reflection and self-evaluation; Other	Implementation outcomes; Cognitive outcomes; Dance learning and performance; Self-related outcomes
Cuellar-Moreno and Caballero-Juliá (2019)	Comparison of Mosston and Ashworth Command Teaching Style versus Problem Solving Teaching Style in dance classes	Each teaching style functioned as the comparator for the other	2 sessions; 60 min per session; 2 weeks; 120 total minutes	Teacher had 15 years of dance experience and postgraduate training/evaluation in Mosston and Ashworth teaching styles	Direct instruction and demonstration; Guided discovery and problem solving	Cognitive outcomes; Motivational-affective outcomes; Psychosocial outcomes
Dryburgh (2019)	Engaged-pedagogy and care-oriented contemporary dance technique teaching emphasizing active participation, student filtering and application of information, reflection on bodily experience, peer stimulation, and teacher responsiveness	No comparator	Not reported	Teacher-researcher had 20 years of experience as a dance artist and teacher and commenced doctoral study during the research period	Autonomy support and learner choice; Reflection and self-evaluation; Feedback and assessment practices; Discussion and verbal sense-making	Dance learning and performance; Self-related outcomes; Psychosocial outcomes; Implementation outcomes
Engdahl et al. (2024)	Creative dance pedagogy based on imitation, mirroring, collaborative planning, discussion, and reflection to open spaces of experimentation and unpredictable movement exploration	No comparator	7 sessions; 90 min per session; 2 weeks; 630 total minutes	Teacher-researcher had worked with dance artistically for 10 years and had taught creative dance for 5 years	Observation and performance comparison; Improvisation and movement exploration; Collaborative learning and peer interaction; Reflection and self-evaluation	Dance learning and performance; Cognitive outcomes; Motivational-affective outcomes; Implementation outcomes
Feltham and Ryan (2022)	Interview-based characterization of pedagogical and recruitment practices for engaging boys in ballet and for including gender non-conforming children, including male role models, media use, same-technique mixed-class teaching, inclusive language, and costume/movement choice	No comparator	Not reported	Participants had between 6 and 15 years of teaching experience; contexts included private studios and a professional training conservatory	Observation and performance comparison; Discussion and verbal sense-making; Direct instruction and demonstration; Autonomy support and learner choice	Participation-related outcomes; Psychosocial outcomes; Implementation outcomes
García-Dantas and Quested (2015)	Comparison of upper-evaluated feedback, objective and informational accurate feedback, lower-evaluated feedback, and no-feedback control during repeated execution of a Zapateado sequence	Upper-evaluated, lower-evaluated, and no-feedback conditions were compared with objective and informational accurate feedback within the same randomized experiment	1 sessions; 60 min per session; 1 weeks; 60 total minutes	Five flamenco teachers from the same conservatory served as confederates who rated performance and provided the basis for manipulated or accurate feedback	Feedback and assessment practices	Self-related outcomes
Guo and Thiagarajan (2025)	Laban/Bartenieff Movement System pedagogy integrating the Body, Effort, Space, and Shape framework, somatic methods, experiential learning, discussion, summarization, and free exploration in ChaoXian dance education	Traditional imitative teaching is the conceptual comparator, but no parallel control group was implemented	11 sessions; 11 weeks	Course instructor had comprehensive Laban/Bartenieff Movement System training and around 20 years of teaching experience	Practice and repetition; Direct instruction and demonstration; Observation and performance comparison; Reflection and self-evaluation; Improvisation and movement exploration	Dance learning and performance; Motor and physical outcomes; Motivational-affective outcomes; Self-related outcomes; Cognitive outcomes; Implementation outcomes
Hsia (2020)	Flipped learning using teacher-recorded instructional videos plus anonymous mobile technology-supported peer assessment during group performance	Flipped learning with the same video-supported preparation but with instructor feedback after group performance instead of peer assessment	8 sessions; 100 min per session; 8 weeks; 800 total minutes	Not reported	Autonomy support and learner choice; Feedback and assessment practices; Observation and performance comparison	Motivational-affective outcomes; Self-related outcomes; Cognitive outcomes; Implementation outcomes
Huddy (2017)	Digitally enhanced progressive assessment using mobile video capture, digital repositories, inquiry, reflective analysis, and oral presentation to build student agency, collaboration, and responsibility for learning	No parallel comparator; the article contrasts the project with conventional teacher-allocated progressive assessment and more didactic technique teaching	Not reported	Dance lecturers; specific qualifications were not reported	Autonomy support and learner choice; Reflection and self-evaluation; Collaborative learning and peer interaction; Feedback and assessment practices	Dance learning and performance; Cognitive outcomes; Self-related outcomes; Psychosocial outcomes; Implementation outcomes
Jin-hee (2010)	Portfolio assessment integrated self-evaluation, peer evaluation, group evaluation, reflective journaling, staged creative tasks, and media-supported review into a contemporary dance creation class	No formal comparator; the article contrasts the portfolio-based class descriptively with more traditional teaching and with midterm/final-only evaluation practices	15 sessions; 120 min per session; 15 weeks; 1,800 total minutes	Not reported	Reflection and self-evaluation; Feedback and assessment practices; Guided discovery and problem solving; Observation and performance comparison; Composition and choreography creation	Dance learning and performance; Motivational-affective outcomes; Self-related outcomes; Cognitive outcomes; Psychosocial outcomes; Implementation outcomes
Jo and Lee (2022)	Teacher participants described group-centered, interest-centered, theme-centered, and skill-centered folk dance teaching-learning content, while also emphasizing the need for stronger systems, teacher expertise, and clearer evaluation standards	No comparator	Not reported	Level 1 or Level 2 teaching certificate holders currently teaching physical education	Collaborative learning and peer interaction; Autonomy support and learner choice; Guided discovery and problem solving; Direct instruction and demonstration	Implementation outcomes; Cognitive outcomes; Psychosocial outcomes
Eun-ju and Dae-jung (2020)	Teacher self-study traced a shift from high-pressure, teacher-centered teaching toward a learner-responsive, relational pedagogy emphasizing student understanding, shared value, dance emotion, and growth guidance	No formal comparator; the self-study contrasted earlier high-pressure teaching with later learner-responsive teaching orientation within the same long-term teaching practice	Not reported	The teacher-researcher reported extensive dance educator experience, including dance academy and company leadership	Autonomy support and learner choice; Collaborative learning and peer interaction; Reflection and self-evaluation	Implementation outcomes; Motivational-affective outcomes; Self-related outcomes; Participation-related outcomes
Jung et al. (2026)	Practice-based Korean dance pedagogy organized as Transmission, Facilitation, and Co-construction	No formal comparator; the study mapped pedagogical practices across authentic educational contexts	Not reported	Experienced dance educators across arts, school physical education, university, and private-instruction settings	Direct instruction and demonstration; Discussion and verbal sense-making; Feedback and assessment practices; Autonomy support and learner choice; Other; Collaborative learning and peer interaction	Dance learning and performance; Self-related outcomes; Cognitive outcomes; Implementation outcomes
Park (2017)	Esthetic-development-based pedagogy for elderly dance education emphasizing body, movement, emotion, and expression	No comparator	Not reported	Dance education experts currently active in elderly dance instruction	Reflection and self-evaluation; Direct instruction and demonstration; Practice and repetition; Observation and performance comparison; Autonomy support and learner choice; Discussion and verbal sense-making; Collaborative learning and peer interaction	Dance learning and performance; Motor and physical outcomes; Motivational-affective outcomes; Psychosocial outcomes; Implementation outcomes
Larsson et al. (2025)	Teacher-guided creative-dance pedagogy using movement-phrase exploration, performance structuring, and the Laban movement analysis framework	No formal comparator; the article contrasts creative-dance practice with more conventional functional and teacher-directed movement traditions	4 sessions; 120 min per session; 480 total minutes	Two highly experienced dance and dance-education teachers co-involved in the study	Composition and choreography creation; Improvisation and movement exploration; Direct instruction and demonstration; Observation and performance comparison; Guided discovery and problem solving	Dance learning and performance; Cognitive outcomes; Self-related outcomes; Implementation outcomes
Longley and Kensington-Miller (2020)	Pedagogy for making invisible graduate attributes explicit through narrative reflection and the SEEN framework	No formal comparator	Not reported	Dance academics and employers informed the framework; exact professional qualifications were not the focus of reporting	Reflection and self-evaluation; Discussion and verbal sense-making; Collaborative learning and peer interaction; Autonomy support and learner choice	Cognitive outcomes; Self-related outcomes; Psychosocial outcomes; Implementation outcomes
Ma (2024)	Online dance-analysis pedagogy combining digital resource retrieval, movement recognition, multimedia support, and multicultural resource integration	Traditional dance teaching	16 weeks	Three teachers scored the final classical-dance assessment; teaching qualifications were not otherwise described	Autonomy support and learner choice; Feedback and assessment practices; Direct instruction and demonstration; Discussion and verbal sense-making; Practice and repetition	Dance learning and performance; Cognitive outcomes
Mabingo (2019)	Experiential and reflective study-abroad pedagogy using local Ugandan dance and music instruction, storytelling, collaborative lesson-planning, co-teaching, and embodied intercultural participation	No comparator	2 weeks	Local Ugandan teachers taught dance, music, and drum-rhythm workshops; specific qualifications were not fully reported	Collaborative learning and peer interaction; Discussion and verbal sense-making; Improvisation and movement exploration; Reflection and self-evaluation; Autonomy support and learner choice	Dance learning and performance; Cognitive outcomes; Psychosocial outcomes; Implementation outcomes
Marani et al. (2022)	Physical-Cultural-Studies-informed dance pedagogy emphasizing democratic dialog, critical reflection, partner exchange, innovative leading practices, and critical analysis of public pedagogies about dance	No formal comparator; the article contrasts critical sociocultural dance teaching with normative and exclusionary gendered practices	Not reported	Dance and physical-education teaching qualifications were discussed autobiographically rather than reported in a standardized way	Collaborative learning and peer interaction; Discussion and verbal sense-making; Reflection and self-evaluation; Guided discovery and problem solving; Autonomy support and learner choice	Cognitive outcomes; Psychosocial outcomes; Self-related outcomes; Implementation outcomes
Mattsson and Larsson (2021)	Teacher-guided expressive-dance pedagogy using exploratory assignments, progressive risk-taking, and Rudolf Laban’s Body, Effort, Space, and Shape framework	No formal comparator; the article contrasts the dance unit with more teacher-centered and pre-determined movement teaching in physical education	8 sessions; 8 weeks	Four licensed physical education teachers; no specific dance training beyond physical education teacher education was reported	Guided discovery and problem solving; Improvisation and movement exploration; Composition and choreography creation; Autonomy support and learner choice; Discussion and verbal sense-making; Other	Dance learning and performance; Cognitive outcomes; Self-related outcomes; Participation-related outcomes; Implementation outcomes
McCarthy-Brown (2021)	Critical dance pedagogy using embodied dialog about race and power, student-generated movement, choreographic creation, reflection, and public performance	No formal comparator	Not reported	The author-facilitator was a dance educator-artist-scholar; formal qualifications were not the focus of reporting	Discussion and verbal sense-making; Composition and choreography creation; Reflection and self-evaluation; Collaborative learning and peer interaction; Autonomy support and learner choice	Cognitive outcomes; Psychosocial outcomes; Motivational-affective outcomes; Self-related outcomes
Mi-Hee (2016)	Autoethnographically reflected teaching trajectory from rigid training and practical-skill instruction toward enjoyable, learner-responsive, culturally meaningful dance education for prospective teachers	No formal comparator; the article contrasts earlier rigid training methods with later more enjoyable and learner-responsive teaching	Not reported	The professor reported long-term dance training from childhood, formal undergraduate and graduate dance education, and extensive university teaching experience	Direct instruction and demonstration; Practice and repetition; Autonomy support and learner choice; Guided discovery and problem solving; Discussion and verbal sense-making	Motivational-affective outcomes; Self-related outcomes; Cognitive outcomes; Implementation outcomes
Jin-kyung et al. (2009)	Predominantly teacher-directed Cecchetti ballet instruction examined through complete demonstration, cueing, and task-focused feedback quality	No formal comparator; the study compared naturally occurring differences in teaching quality across four teachers	10 sessions; 50 min per session; 5 weeks; 500 total minutes	All four teachers held international Cecchetti junior-teacher certification; mean teaching experience was 5.75 years	Direct instruction and demonstration; Feedback and assessment practices	Dance learning and performance; Implementation outcomes
Ørbæk and Engelsrud (2021)	Creative-dance pedagogy evolving from set-movement instruction toward expressive exploration and movement-emotion-language connections	No formal comparator; the article contrasts set-movement instruction with more exploratory, interaction-sensitive teaching	Not reported	Participants were physical education teacher education students completing practicum in Norwegian schools	Direct instruction and demonstration; Improvisation and movement exploration; Autonomy support and learner choice; Discussion and verbal sense-making	Dance learning and performance; Cognitive outcomes; Self-related outcomes; Implementation outcomes
Park (2017)	Esthetic-experience-oriented dance education emphasizing imagination, expression, creation, appreciation, varied stimuli, autonomous methods, and group activity; the study derived the Ae-SD strategy	No formal comparator; the article contrasts esthetic-experience-oriented pedagogy with technical, repetitive, and expression-poor dance learning	Not reported	The program was supported by the Seoul Foundation for Arts and Culture; teacher credentials were not the focus of reporting	Autonomy support and learner choice; Composition and choreography creation; Observation and performance comparison; Collaborative learning and peer interaction; Improvisation and movement exploration	Motivational-affective outcomes; Self-related outcomes; Psychosocial outcomes; Implementation outcomes
Powell (2025)	Feminist and democratic dance-technique pedagogy using open questions, discussion, shared decision-making, collaboration, and differentiation to challenge authoritarian teaching	No formal comparator; the article contrasts feminist-democratic practice with traditional authoritarian demonstration-instruction-correction methods	Not reported	The author had worked for 6 years as a Cunningham-based contemporary dance tutor at the specialist higher-education ballet school	Discussion and verbal sense-making; Autonomy support and learner choice; Collaborative learning and peer interaction; Guided discovery and problem solving	Self-related outcomes; Cognitive outcomes; Psychosocial outcomes; Implementation outcomes
Rimmer (2017)	Enquiry-based, constructivist dance-technique pedagogy using partner problem-solving, phrase creation, improvisation, discussion, and journal reflection to support active learner agency	No formal comparator; the article contrasts enquiry-based learning with traditional right-wrong, teacher-led dance-technique expectations	11 sessions; 11 weeks	The researcher was a higher-education dance-technique teacher; colleague A mirrored some enquiry-based approaches in a parallel cohort	Guided discovery and problem solving; Collaborative learning and peer interaction; Composition and choreography creation; Improvisation and movement exploration; Reflection and self-evaluation	Cognitive outcomes; Self-related outcomes; Implementation outcomes; Participation-related outcomes
Rimmer-Piekarczyk (2018)	Dialogic, somatic-informed teaching that returned questions to students, foregrounded first-person reflection, used exploratory tasks, peer observation, peer feedback, improvisation, and collaborative discussion to challenge hierarchical technique teaching.	No separate comparator arm; comparison is implicit against more traditional question-answer and authoritative technique teaching.	8 sessions; 9 weeks	Teacher-researcher delivering first-year higher education dance technique; exact credentials not reported.	Guided discovery and problem solving; Improvisation and movement exploration; Collaborative learning and peer interaction; Discussion and verbal sense-making	Self-related outcomes; Cognitive outcomes; Psychosocial outcomes; Implementation outcomes
Rothmund (2023)	The study analyzed student learning experiences across a continuum from teacher-centered reproductive technique work toward more student-centered explorative, improvisational, and self-regulated learning practices.	No formal comparator arm; the analytic comparison is between more reproductive and more explorative teaching approaches within the program	Not reported	Program dance teachers; individual qualifications not reported in article.	Practice and repetition; Guided discovery and problem solving; Improvisation and movement exploration; Reflection and self-evaluation	Dance learning and performance; Self-related outcomes; Psychosocial outcomes; Motivational-affective outcomes; Implementation outcomes
Soerel et al. (2023)	The study mapped naturally occurring instruction, questioning, feedback, and focus-of-attention practices to characterize how teaching information was delivered across class types.	Natural comparison across different classes, rehearsals, and teaching modes rather than assigned intervention groups.	8 sessions; 77 min per session; 8 weeks; 618 total minutes	All six teachers had more than 10 years of dance-teaching experience.	Direct instruction and demonstration; Feedback and assessment practices; Discussion and verbal sense-making; Guided discovery and problem solving	Implementation outcomes
de Garcías Ves and Beltran-Garrido (2024)	Specialized body-percussion pedagogy integrated into Body Expression and Dance, using rhythmic motor games, cooperative participation, rotating artistic roles, and musical improvisation to explore body-time relations	No comparator; within-group pre- and post-program repeated-measures comparison by sex	Not reported	Researcher-instructor was a course teacher and body-percussion specialist with professional and teacher-training experience	Collaborative learning and peer interaction; Autonomy support and learner choice; Improvisation and movement exploration; Direct instruction and demonstration	Motivational-affective outcomes
Vincent et al. (2021)	The study compared teacher-reported delivery of certificated dance curricula, highlighting a more integrated, resource-based and partially student-centered GCSE approach versus more compartmentalized National 5 delivery with variable discovery learning	Comparative contrast between English GCSE Dance and Scottish National 5 Dance curricular delivery contexts	Not reported	England participants generally held undergraduate dance degrees and secondary-dance teaching credentials, whereas Scotland participants mainly held physical-education degrees or postgraduate physical-education teaching qualifications with variable dance backgrounds	Observation and performance comparison; Composition and choreography creation; Discussion and verbal sense-making; Collaborative learning and peer interaction; Direct instruction and demonstration; Guided discovery and problem solving	Implementation outcomes; Psychosocial outcomes; Self-related outcomes; Cognitive outcomes
Wilson (2009)	Somatic experiential-anatomy pedagogy that presented anatomical and kinesiological concepts and then asked dancers to explore, embody, journal, discuss, and apply them in movement and technique contexts	No formal comparator; the inquiry examined how combined academic and experiential somatic learning operated across multiple cases	Not reported	Teacher-researcher was a university dance educator; one graduate experiential-anatomy component was co-taught with a colleague	Direct instruction and demonstration; Improvisation and movement exploration; Reflection and self-evaluation; Discussion and verbal sense-making	Cognitive outcomes; Self-related outcomes; Dance learning and performance; Motor and physical outcomes; Implementation outcomes

### 3.5 Results of individual studies

Twenty-one studies reported direct dance learning or performance outcomes (Table 7). However, only five studies reported motor and physical outcomes, and the evidence was almost entirely descriptive. Fourteen studies addressed motivational-affective outcomes (Table 8). Self-related outcomes were among the most densely represented domains. Cognitive outcomes were reported in 24 studies and constituted one of the clearest patterns in the review (Table 9). Psychosocial outcomes were reported in 17 studies and were concentrated around collaborative learning, relational trust, empathy, and social meaning-making. Participation-related outcomes were reported in only four studies and remain markedly underdeveloped.

**Table 7:** Individual-study findings for dance learning and performance and motor and physical outcomes.

Study	Pedagogical approach or intervention	Outcome domain(s)	Main findings
Jae-im (2013)	Naturalistic creative dance teaching with warm-up, free movement exploration, improvisational and technical skill development, discussion, creating and sharing, collaborative work, and self and group reflection	Dance learning and performance; Motor and physical outcomes	Original movement studies were created as solos, duets, and groups and performed during informal showings for the class. Goals of the dance class included kinesthetic awareness, control, balance, and coordination.
Andersson (2018)	Naturalistic assessment and teaching practices in dance education, including teacher demonstration, communicated feedback, student independence in performance, comparison of performances, and varied modes of embodiment	Dance learning and performance	Teachers indicated that assessment requires students to perform the material independently and not rely completely on the teacher as visual support.
Berg (2017)	IMAGE TECH for Dancers (ITD) integrated Alexander Technique, Irene Dowd’s neuromuscular retraining/ideokinesis, tactile cueing, imagery, anatomical dialog, and self-correction into ballet pedagogy	Dance learning and performance; Motor and physical outcomes	Somatic integration supported technique learning by helping dancers adapt ballet work to their own structure rather than reproduce a single externally prescribed ideal. The somatic approach was described as helping dancers find safer alignment and more sustainable technical organization, including injury-prevention or recovery-oriented learning.
Brown (2023)	Student-centered technique pedagogy incorporating peer feedback, student-led teaching, video self-review with student-created rubrics, and student-run discussion/Socratic seminar	Dance learning and performance	Direct-instruction groups showed statistically significant pre/post improvement in most rubric areas, and in the non-magnet sample direct instruction improved as much as or more than the student-centered condition. Student-centered groups improved significantly on most rubric areas, but ANCOVA did not show that the intervention was more successful than direct instruction.
Chen (2025)	Technology-enhanced dance education program using Presentation Creator, Flipgrid, Dancy, Video Traces, and Dance Designer to support visualization, targeted teacher feedback, rehearsal, and independent study	Dance learning and performance	Both groups achieved high effectiveness ratings after the digital teacher-feedback program; Group 1 was described as more precise/traditional and Group 2 as more flexible in jumps. Most students achieved excellent or minor-error Ribbon Dance performance when learning independently through digitally mediated guidance and feedback.
Choi and Kim (2015)	Whole-ballet pedagogy framing ballet education across physical, cognitive, emotional, and spiritual dimensions, with ten direct and indirect teaching methods identified	Dance learning and performance	The whole-ballet framework argued that technical and artistic development should be integrated through complementary direct and indirect methods rather than technique-only teaching.
Dryburgh (2019)	Engaged-pedagogy and care-oriented contemporary dance technique teaching emphasizing active participation, student filtering and application of information, reflection on bodily experience, peer stimulation, and teacher responsiveness	Dance learning and performance	Students described technique learning as partnership, application, filtering, and trial and error, suggesting that active participation supported meaningful technique development.
Dryburgh (2019)	Creative dance pedagogy based on imitation, mirroring, collaborative planning, discussion, and reflection to open spaces of experimentation and unpredictable movement exploration	Dance learning and performance	The pedagogical sequence suggested that students can learn to operate with ambiguous and unexpected movement experiences rather than only adopt predetermined forms of dance.
Engdahl et al. (2024)	Laban/Bartenieff Movement System pedagogy integrating the Body, Effort, Space, and Shape framework, somatic methods, experiential learning, discussion, summarization, and free exploration in ChaoXian dance education	Dance learning and performance; Motor and physical outcomes	The Body, Effort, and Shape components were reported to improve fluidity, stability, precision, and expressiveness in ChaoXian movement execution. Students reported heightened bodily awareness, improved connectivity, and better control of movement through breath work, body-organization patterns, and Basic Six exercises.
Huddy (2017)	Digitally enhanced progressive assessment using mobile video capture, digital repositories, inquiry, reflective analysis, and oral presentation to build student agency, collaboration, and responsibility for learning	Dance learning and performance	Students described learning to experiment with ways of making technique fit their body, indicating a more adaptive and embodied approach to technique learning.
Jin-hee (2010)	Portfolio assessment integrated self-evaluation, peer evaluation, group evaluation, reflective journaling, staged creative tasks, and media-supported review into a contemporary dance creation class	Dance learning and performance	The staged portfolio-based class was reported to help students move from uncertainty toward more original movement production, more deliberate composition, and stronger creative understanding.
Jo and Lee (2022)	Practice-based Korean dance pedagogy organized as Transmission, Facilitation, and Co-construction	Dance learning and performance	The typology was presented as a way to scaffold embodied learning by linking explanation, individualized guidance, facilitation, and co-construction in real teaching situations.
Eun-ju and Dae-jung (2020)	Esthetic-development-based pedagogy for elderly dance education emphasizing body, movement, emotion, and expression	Motor and physical outcomes; Dance learning and performance	Experts emphasized body awareness, breathing, and pace control as crucial for safe movement and prevention of strain or injury in elderly dance education. Repeated practice, tools, and performance experience were described as methods that help older learners become familiar with movement, extend expression, and continue participating in dance.
Larsson et al. (2025)	Teacher-guided creative-dance pedagogy using movement-phrase exploration, performance structuring, and the Laban movement analysis framework	Dance learning and performance	Students were reported to practice making sense of moving in non-predetermined creative ways and to refine movement phrases toward stronger expression, flow, and simultaneity.
Ma (2024)	Online dance-analysis pedagogy combining digital resource retrieval, movement recognition, multimedia support, and multicultural resource integration	Dance learning and performance	The online dance-analysis teaching method produced more students in the excellent and good categories and fewer in the passing and failing categories than traditional teaching.
Mabingo (2019)	Experiential and reflective study-abroad pedagogy using local Ugandan dance and music instruction, storytelling, collaborative lesson-planning, co-teaching, and embodied intercultural participation	Dance learning and performance	Students described the program as facilitating holistic learning of neo-traditional dance through music and storytelling rather than through isolated movement reproduction.
Mattsson and Larsson (2021)	Teacher-guided expressive-dance pedagogy using exploratory assignments, progressive risk-taking, and Rudolf Laban’s Body, Effort, Space, and Shape framework	Dance learning and performance	Expanded transactions occurred when students found their own solutions to dance assignments, and the culminating choreographies showed new expressive ways of moving.
Jin-kyung et al. (2009)	Predominantly teacher-directed Cecchetti ballet instruction examined through complete demonstration, cueing, and task-focused feedback quality	Dance learning and performance	Students taught by the teacher with the highest Qualitative Measures of Teaching Performance Scale score showed the greatest Pirouettes improvement, indicating that stronger task presentation and feedback were associated with better skill learning. Arabesque improvement followed the same teacher-quality pattern, with better Qualitative Measures of Teaching Performance Scale performance aligning with greater learner improvement.
Ørbæk and Engelsrud (2021)	Creative-dance pedagogy evolving from set-movement instruction toward expressive exploration and movement-emotion-language connections	Dance learning and performance	Teacher students described pupils as moving more freely when the teachers physically stepped back at the start of the creative process.
Rothmund (2023)	The study analyzed student learning experiences across a continuum from teacher-centered reproductive technique work toward more student-centered explorative, improvisational, and self-regulated learning practices.	Dance learning and performance	Students described learning processes that combined mastering set material with understanding principles and adapting them to their own bodies.
Wilson (2009)	Somatic experiential-anatomy pedagogy that presented anatomical and kinesiological concepts and then asked dancers to explore, embody, journal, discuss, and apply them in movement and technique contexts	Dance learning and performance; Motor and physical outcomes	The final synthesis argued that multiple modes of embodiment helped move understanding from isolated movement exploration into actual dancing and technique practice. An axial-coding example described a dancer with leg-length discrepancy and pain who reported that the Experiential Anatomy class gave her ideas for dealing with the pain.

**Table 8:** Individual-study findings for motivational-affective outcomes and self-related outcomes.

Study	Pedagogical approach or intervention	Outcome domain(s)	Main findings
Andersson (2018)	Naturalistic assessment and teaching practices in dance education, including teacher demonstration, communicated feedback, student independence in performance, comparison of performances, and varied modes of embodiment	Motivational-affective outcomes	Student motivation and responsibility for learning in dance emerged as one aspect of conditions for assessment for learning.
Berg (2017)	IMAGE TECH for Dancers (ITD) integrated Alexander Technique, Irene Dowd’s neuromuscular retraining/ideokinesis, tactile cueing, imagery, anatomical dialog, and self-correction into ballet pedagogy	Self-related outcomes	Students were encouraged to become self-correcting dancers who could interpret and act on internal sensations rather than rely only on teacher authority. The pedagogical approach emphasized perceiving what technique felt like internally and using that awareness to organize movement.
Brown (2023)	Student-centered technique pedagogy incorporating peer feedback, student-led teaching, video self-review with student-created rubrics, and student-run discussion/Socratic seminar	Self-related outcomes	Students in the intervention condition reported lower comfort with peer feedback after the study, whereas direct-instruction participants generally became more positive about class.
Chen (2025)	Technology-enhanced dance education program using Presentation Creator, Flipgrid, Dancy, Video Traces, and Dance Designer to support visualization, targeted teacher feedback, rehearsal, and independent study	Motivational-affective outcomes	Most students in both groups reported positive attitudes toward dance learning with digital technologies and educator interaction.
Choi and Kim (2015)	Whole-ballet pedagogy framing ballet education across physical, cognitive, emotional, and spiritual dimensions, with ten direct and indirect teaching methods identified	Self-related outcomes	Indirect methods such as reflective stimulation and character transference were framed as important for developing autonomy, values, and individual artistry in ballet learning.
Cuellar-Moreno and Caballero-Juliá (2019)	Comparison of Mosston and Ashworth Command Teaching Style versus Problem Solving Teaching Style in dance classes	Motivational-affective outcomes	Most students preferred the Problem Solving teaching style after experiencing both lessons. Students who preferred Command style said it felt easier and more secure because they could copy a model and maintain rhythm, especially when they lacked practice or confidence.
Dryburgh (2019)	Engaged-pedagogy and care-oriented contemporary dance technique teaching emphasizing active participation, student filtering and application of information, reflection on bodily experience, peer stimulation, and teacher responsiveness	Self-related outcomes	The pedagogy was interpreted as supporting active learner identity, personal meaning-making, and less reliance on the teacher as students learned to apply information in personally meaningful ways.
Engdahl et al. (2024)	Creative dance pedagogy based on imitation, mirroring, collaborative planning, discussion, and reflection to open spaces of experimentation and unpredictable movement exploration	Motivational-affective outcomes	Students reported feeling freedom and excitement when given possibilities to explore because the movements were unpredictable and not predetermined.
García-Dantas and Quested (2015)	Comparison of upper-evaluated feedback, objective and informational accurate feedback, lower-evaluated feedback, and no-feedback control during repeated execution of a Zapateado sequence	Self-related outcomes	There was no statistically significant between-group difference in post-manipulation self-efficacy across the four feedback conditions. Learners in the upper-evaluated condition showed a statistically significant increase in self-efficacy from pre- to post-manipulation. Accurate and informational feedback produced a statistically significant increase in self-efficacy and the largest proportion of learners with increased self-efficacy.
Guo and Thiagarajan (2025)	Laban/Bartenieff Movement System pedagogy integrating the Body, Effort, Space, and Shape framework, somatic methods, experiential learning, discussion, summarization, and free exploration in ChaoXian dance education	Self-related outcomes; Motivational-affective outcomes	Students described increased somatic authority, greater control over their movement, and more autonomous, self-guided approaches to learning. Students reported comfort, relaxation, pleasure, enjoyment, and satisfaction from exploratory somatic work and from solving movement problems independently.
Hsia (2020)	Flipped learning using teacher-recorded instructional videos plus anonymous mobile technology-supported peer assessment during group performance	Motivational-affective outcomes; Self-related outcomes	Students in the flipped learning plus mobile peer-assessment condition showed significantly higher adjusted intrinsic motivation than students in the flipped learning plus instructor-feedback condition. There was no statistically significant difference in adjusted extrinsic motivation between the mobile peer-assessment and instructor-feedback flipped classes. There was no statistically significant difference in self-efficacy between the.
Huddy (2017)	Digitally enhanced progressive assessment using mobile video capture, digital repositories, inquiry, reflective analysis, and oral presentation to build student agency, collaboration, and responsibility for learning	Self-related outcomes	The project was reported to strengthen student agency by placing learners in active roles as investigators, collaborators, and contributors to their own technique development.
Jin-hee (2010)	Portfolio assessment integrated self-evaluation, peer evaluation, group evaluation, reflective journaling, staged creative tasks, and media-supported review into a contemporary dance creation class	Self-related outcomes; Motivational-affective outcomes	Students reported that portfolio assessment helped them gain confidence in creative dance, reduced early embarrassment, and encouraged them to attempt more expressive and original movement. Students described the class as increasingly interesting and enjoyable and said they wanted similar portfolio-based evaluation in future courses.
Jo and Lee (2022)	Teacher self-study traced a shift from high-pressure, teacher-centered teaching toward a learner-responsive, relational pedagogy emphasizing student understanding, shared value, dance emotion, and growth guidance	Motivational-affective outcomes; Self-related outcomes	The teacher-researcher argued that understanding students and shifting away from pressure helped address learner unfamiliarity and negative preconceptions about dance. The revised teaching orientation emphasized cultivating dance emotion and guiding student growth, indicating a more developmental and self-supportive understanding of liberal dance pedagogy.
Jung et al. (2026)	Practice-based Korean dance pedagogy organized as Transmission, Facilitation, and Co-construction	Self-related outcomes	Practices such as encouraging learners’ own interpretations, respectful relationships, and patient encouragement were described as important for expression and sustained engagement.
Park (2017)	Esthetic-development-based pedagogy for elderly dance education emphasizing body, movement, emotion, and expression	Motivational-affective outcomes	Performance experiences were described as major motivators that gave older learners confidence, a sense of achievement, and reason to continue practicing and participating.
Larsson et al. (2025)	Teacher-guided creative-dance pedagogy using movement-phrase exploration, performance structuring, and the Laban movement analysis framework	Self-related outcomes	Students described caring about the dance differently when they created and explored movement themselves, and the analysis linked agency to stronger, more heartfelt involvement.
Longley and Kensington-Miller (2020)	Pedagogy for making invisible graduate attributes explicit through narrative reflection and the SEEN framework	Self-related outcomes	The narratives emphasized resilience, negotiating skills, adaptability, and broader life skills as important outcomes that tertiary dance pedagogy should help students recognize and carry forward.
Marani et al. (2022)	Physical-Cultural-Studies-informed dance pedagogy emphasizing democratic dialog, critical reflection, partner exchange, innovative leading practices, and critical analysis of public pedagogies about dance	Self-related outcomes	The professor described classroom situations in which discomfort, refusal, and withdrawal around dance interactions became pedagogical sites for rethinking how students relate to dance and to themselves.
Mattsson and Larsson (2021)	Teacher-guided expressive-dance pedagogy using exploratory assignments, progressive risk-taking, and Rudolf Laban’s Body, Effort, Space, and Shape framework	Self-related outcomes	Expanded transactions were most evident when students were given the opportunity and responsibility to find their own solutions to dance assignments.
McCarthy-Brown (2021)	Critical dance pedagogy using embodied dialog about race and power, student-generated movement, choreographic creation, reflection, and public performance	Motivational-affective outcomes; Self-related outcomes	The study documented denial, silence, uncertainty, and apprehension, especially among some White students, showing that discomfort was a central part of the process. The article stated that the embodied learning experiences proved impactful for over 90 % of participants and that almost all participants cited educational growth.
Mi-Hee (2016)	Autoethnographically reflected teaching trajectory from rigid training and practical-skill instruction toward enjoyable, learner-responsive, culturally meaningful dance education for prospective teachers	Motivational-affective outcomes; Self-related outcomes	Later teaching approaches were described as enjoyable and easier to understand, helping students reduce discomfort and experience dance class as fun. The class was described as prompting prospective teachers to become more interested in their own bodies and to develop more positive perceptions of dance.
Ørbæk and Engelsrud (2021)	Creative-dance pedagogy evolving from set-movement instruction toward expressive exploration and movement-emotion-language connections	Self-related outcomes	Teacher students described becoming more aware of how their own positioning and interaction shaped pupils’ responses in creative dance.
Park (2017)	Esthetic-experience-oriented dance education emphasizing imagination, expression, creation, appreciation, varied stimuli, autonomous methods, and group activity; the study derived the Ae-SD strategy	Motivational-affective outcomes; Self-related outcomes	Learners described esthetic experience through imagining, sensing, and expressive engagement rather than through technical mastery alone. Participants described opening their minds, seeing newly, and voicing themselves more readily through the program.
Powell (2025)	Feminist and democratic dance-technique pedagogy using open questions, discussion, shared decision-making, collaboration, and differentiation to challenge authoritarian teaching	Self-related outcomes	The article argued that feminist strategies such as open questions and shared decision-making can support student voice, agency, and empowerment in technique learning.
Rimmer (2017)	Enquiry-based, constructivist dance-technique pedagogy using partner problem-solving, phrase creation, improvisation, discussion, and journal reflection to support active learner agency	Self-related outcomes	Students’ prior expectations about being right and relying on the teacher made open-ended technique learning feel uncertain and demanding.
Rimmer-Piekarczyk (2018)	Dialogic, somatic-informed teaching that returned questions to students, foregrounded first-person reflection, used exploratory tasks, peer observation, peer feedback, improvisation, and collaborative discussion to challenge hierarchical technique teaching.	Self-related outcomes	Data analysis suggested that dialogic reflection and body listening supported the development of self-somatic authority for some students. The pedagogy appeared to shift responsibility toward learners and encouraged them to attend to their own bodies and learning processes.
Rothmund (2023)	The study analyzed student learning experiences across a continuum from teacher-centered reproductive technique work toward more student-centered explorative, improvisational, and self-regulated learning practices.	Self-related outcomes; Motivational-affective outcomes	Over time students became more self-regulated and took more responsibility for their own learning processes. Students were described as becoming more interested in student-centered approaches as their education progressed.
de Garcías Ves and Beltran-Garrido (2024)	Specialized body-percussion pedagogy integrated into Body Expression and Dance, using rhythmic motor games, cooperative participation, rotating artistic roles, and musical improvisation to explore body-time relations	Motivational-affective outcomes	Overall mood scores improved from pre- to post-program in both male and female participants, with no clear sex-by-time interaction. Tension scores changed little, and planned contrasts did not show significant pre-post change within either sex despite the overall time model reported in the paper. Depression scores decreased significantly from pre- to post-program in both male and female participants. Anger scores decreased significantly in.
Wilson (2009)	Somatic experiential-anatomy pedagogy that presented anatomical and kinesiological concepts and then asked dancers to explore, embody, journal, discuss, and apply them in movement and technique contexts	Self-related outcomes	One of the central themes was that dancers worked with information about the body through the body itself, indicating increased bodily awareness and embodied self-relation.

**Table 9:** Individual-study findings for cognitive, psychosocial, participation-related, and implementation outcomes.

Study	Pedagogical approach or intervention	Outcome domain(s)	Main findings
Jae-im (2013)	Naturalistic creative dance teaching with warm-up, free movement exploration, improvisational and technical skill development, discussion, creating and sharing, collaborative work, and self and group reflection	Cognitive outcomes; Psychosocial outcomes	The class focused on building cognitive skills through problem solving, critical thinking, conceptualization, and articulation of ideas. To build social skills and a spirit of mutual respect and cooperation, the class used collaborative work, observation, and self and group reflection.
Andersson (2018)	Naturalistic assessment and teaching practices in dance education, including teacher demonstration, communicated feedback, student independence in performance, comparison of performances, and varied modes of embodiment	Implementation outcomes; Psychosocial outcomes	Two major themes emerged: conditions for assessment for learning and making space for assessment. The meaning of communicated feedback and students’ various needs and abilities were described as essential aspects of assessment for learning.
Brown (2023)	Student-centered technique pedagogy incorporating peer feedback, student-led teaching, video self-review with student-created rubrics, and student-run discussion/Socratic seminar	Cognitive outcomes	Although technical gains were not superior, the author argued that student-centered practices may have enabled more viewing, feedback, articulation, and higher-order thinking about dance.
Chen (2025)	Technology-enhanced dance education program using Presentation Creator, Flipgrid, Dancy, Video Traces, and Dance Designer to support visualization, targeted teacher feedback, rehearsal, and independent study	Implementation outcomes	The program emphasized communication, dialog, time management, and collaborative creative activity as key criteria for effective teacher-student interaction in digital dance education.
Choi and Kim (2015)	Whole-ballet pedagogy framing ballet education across physical, cognitive, emotional, and spiritual dimensions, with ten direct and indirect teaching methods identified	Implementation outcomes; Cognitive outcomes	The study distinguished direct and indirect methods and argued that both are needed, with environmental and contextual features such as milieu creation functioning as important parts of pedagogy. Indirect methods such as experience recommendation and dialogic explanation were presented as useful for developing contextual and cultural understanding of ballet.
Cuellar-Moreno and Caballero-Juliá (2019)	Comparison of Mosston and Ashworth Command Teaching Style versus Problem Solving Teaching Style in dance classes	Cognitive outcomes	Cognitive participation was significantly greater in the Problem Solving teaching style than in the Command teaching style. Interview coding showed a wider range of Bloom-taxonomy cognitive categories, creativity, and mental effort under Problem Solving than under Command style.
Dryburgh (2019)	Engaged-pedagogy and care-oriented contemporary dance technique teaching emphasizing active participation, student filtering and application of information, reflection on bodily experience, peer stimulation, and teacher responsiveness	Psychosocial outcomes; Implementation outcomes	Students linked feeling valued and teachers being present, approachable, and constructively exacting with greater willingness to engage and learn, whereas exclusionary teaching inhibited learning. The study organized findings around three enabling pedagogical conditions: active participation, demonstration of value and care, and stimulating peer environments.
Engdahl et al. (2024)	Creative dance pedagogy based on imitation, mirroring, collaborative planning, discussion, and reflection to open spaces of experimentation and unpredictable movement exploration	Cognitive outcomes; Implementation outcomes	Students described creative dance as moving in spaces of unpredictability where movements were not known in advance and where exploration rather than pre-planned mastery became central. Exploratory outcomes were linked to improvised movement, presentation of movement in the moment, removal of evaluative pressure, and iterative revision of tasks with students.
Feltham and Ryan (2022)	Interview-based characterization of pedagogical and recruitment practices for engaging boys in ballet and for including gender non-conforming children, including male role models, media use, same-technique mixed-class teaching, inclusive language, and costume/movement choice	Participation-related outcomes; Psychosocial outcomes; Implementation outcomes	Teachers reported that welcoming environments, role models, and strategic media use can help engage boys, but participation remains constrained by stigma and parent beliefs, and male-focused strategies often remain hypermasculine. Instructors emphasized that boys should feel they belong in ballet and that inclusive language, options, and representation can help boys and gender non-conforming children feel welcome and respected. Teachers.
Guo and Thiagarajan (2025)	Laban/Bartenieff Movement System pedagogy integrating the Body, Effort, Space, and Shape framework, somatic methods, experiential learning, discussion, summarization, and free exploration in ChaoXian dance education	Cognitive outcomes; Implementation outcomes	BESS changed students’ movement cognition, improved analytical precision, supported independent problem-solving, and fostered creativity in choreography and transfer beyond class. Students initially struggled with closed-eye work, free exploration, and inward attention because of habits of seeking external correctness and conformity, although discomfort reduced over time.
Hsia (2020)	Flipped learning using teacher-recorded instructional videos plus anonymous mobile technology-supported peer assessment during group performance	Cognitive outcomes; Implementation outcomes	Students reported that the mobile peer-assessment activity increased concentration, careful observation of others’ dancing, and self-reflection through self-other comparison. Students described the flipped videos as helpful for self-paced practice, deeper observation of movement detail, and self-correction without needing to depend on classmates.
Huddy (2017)	Digitally enhanced progressive assessment using mobile video capture, digital repositories, inquiry, reflective analysis, and oral presentation to build student agency, collaboration, and responsibility for learning	Psychosocial outcomes; Cognitive outcomes; Implementation outcomes	The digitally enhanced pedagogy was reported to foster peer learning, trust, empathy, and a marked culture shift toward more open collaborative engagement in the technique studio. The digitally enhanced pedagogy was reported to improve critical reflection, problem solving, and independent inquiry by making students review footage, analyze their learning, and articulate evidence of progression.
Jin-hee (2010)	Portfolio assessment integrated self-evaluation, peer evaluation, group evaluation, reflective journaling, staged creative tasks, and media-supported review into a contemporary dance creation class	Participation-related outcomes; Psychosocial outcomes; Cognitive outcomes	Portfolio assessment was reported to make students participate more actively and to improve the efficiency of learning through continuous engagement with tasks and evaluation. Portfolio-based group work promoted cooperation, mutual support, idea exchange, feedback, and stronger interaction among students and between students and the teacher.
Jo and Lee (2022)	Teacher participants described group-centered, interest-centered, theme-centered, and skill-centered folk dance teaching-learning content, while also emphasizing the need for stronger systems, teacher expertise, and clearer evaluation standards	Cognitive outcomes; Psychosocial outcomes; Implementation outcomes	Teachers perceived folk dance as educationally valuable for creativity, new value creation, cultural identity, and recognition of distinctive cultural heritage. Teachers associated folk dance with expressive development, emotional and character education, and social outcomes such as cooperation and connectedness.
Eun-ju and Dae-jung (2020)	Teacher self-study traced a shift from high-pressure, teacher-centered teaching toward a learner-responsive, relational pedagogy emphasizing student understanding, shared value, dance emotion, and growth guidance	Implementation outcomes	The self-study described a clear pedagogical shift away from coercive, pressure-based teaching toward learner-responsive teaching built on student understanding and support. The article concluded that continuous self-study by dance instructors and more open professional learning environments are important for improving the quality of college dance classes.
Jung et al. (2026)	Practice-based Korean dance pedagogy organized as Transmission, Facilitation, and Co-construction	Implementation outcomes; Cognitive outcomes	The study identified fifteen core practices of Korean dance and organized them into the pedagogical types of Transmission, Facilitation, and Co-construction as a flexible continuum. The framework emphasized explanation of movement characteristics, music, emotion, and cultural-historical context as core parts of Korean dance learning.
Park (2017)	Esthetic-development-based pedagogy for elderly dance education emphasizing body, movement, emotion, and expression	Psychosocial outcomes; Implementation outcomes	Self-presentation, narrative sharing, and ritual-community building were described as ways older learners express themselves, communicate, and form caring educational communities through dance. The article proposed a new direction for elderly dance education by organizing pedagogical methods around esthetic development rather than only health or therapy.
Larsson et al. (2025)	Teacher-guided creative-dance pedagogy using movement-phrase exploration, performance structuring, and the Laban movement analysis framework	Cognitive outcomes; Implementation outcomes	The study argued that students learned to appreciate expressive dimensions of movement rather than focus only on functional correctness. The Laban movement analysis framework, combined with knowledgeable teacher demonstration, was described as providing a clear yet permissive structure that enabled exploration beyond habitual movement patterns.
Longley and Kensington-Miller (2020)	Pedagogy for making invisible graduate attributes explicit through narrative reflection and the SEEN framework	Cognitive outcomes; Psychosocial outcomes; Implementation outcomes	The article argued that dance students develop many important attributes that are often invisible on transcripts and that these can be more explicitly recognized and articulated through pedagogy. The SEEN framework example and the narratives highlighted cultural awareness, empathy, care, collaboration, leadership, and respect as important dance-education outcomes.
Ma (2024)	Online dance-analysis pedagogy combining digital resource retrieval, movement recognition, multimedia support, and multicultural resource integration	Cognitive outcomes	The article concluded that the online dance-analysis method improved students’ mastery of classical-dance theory knowledge, dance techniques, and combination movements compared with traditional teaching.
Mabingo (2019)	Experiential and reflective study-abroad pedagogy using local Ugandan dance and music instruction, storytelling, collaborative lesson-planning, co-teaching, and embodied intercultural participation	Cognitive outcomes; Psychosocial outcomes; Implementation outcomes	Students negotiated, questioned, and conceptualized Uganda as a place of difference, exoticism, and identity variation through embodied study-abroad participation. The program was described as cultivating embodied connections to the “other” through collaborative and communalized pedagogical participation.
Marani et al. (2022)	Physical-Cultural-Studies-informed dance pedagogy emphasizing democratic dialog, critical reflection, partner exchange, innovative leading practices, and critical analysis of public pedagogies about dance	Cognitive outcomes; Psychosocial outcomes; Implementation outcomes	The dance-focused narratives described how students were directed to understand dance in physical education as socially, culturally, and politically produced through power relations, especially gender and sexuality. The pedagogy sought to make the lives and dance experiences of gender and sexual minorities more possible and more supportable, valuing bodies beyond compulsory norms.
Mattsson and Larsson (2021)	Teacher-guided expressive-dance pedagogy using exploratory assignments, progressive risk-taking, and Rudolf Laban’s Body, Effort, Space, and Shape framework	Cognitive outcomes; Participation-related outcomes; Implementation outcomes	The students were described as developing new ways of expressing themselves and as focusing more on the meaning of their movements. Interrupted transactions were observed when students hesitated or avoided participation, indicating that risk could both open and constrain expressive engagement. Teaching methods involving a degree of risk, together with unfamiliar music, dimmed lighting, separate rooms, and the Laban framework, were described as.
McCarthy-Brown (2021)	Critical dance pedagogy using embodied dialog about race and power, student-generated movement, choreographic creation, reflection, and public performance	Cognitive outcomes; Psychosocial outcomes	Students were required to investigate race-based systems of oppression, and the study reported substantial educational growth in critical understanding across the embodied dialog process. The article highlighted the value of embodied dialog, indicating that the choreography process opened relational learning spaces around race and difference.
Mi-Hee (2016)	Autoethnographically reflected teaching trajectory from rigid training and practical-skill instruction toward enjoyable, learner-responsive, culturally meaningful dance education for prospective teachers	Cognitive outcomes; Implementation outcomes	The professor reported that students began to discover new meanings in dance and that prospective elementary teachers learned artistic creativity and imagination as important qualities. The self-study critically reflected on tensions between rigid art training, the physical-education context, and the need for more learner-responsive dance pedagogy in teacher education.
Jin-kyung et al. (2009)	Predominantly teacher-directed Cecchetti ballet instruction examined through complete demonstration, cueing, and task-focused feedback quality	Implementation outcomes	Higher total Qualitative Measures of Teaching Performance Scale scores were interpreted as evidence of more effective teaching and aligned with higher student achievement. Overall cueing and congruent feedback were reported as lower than desirable, suggesting a practical teaching-quality gap even within otherwise teacher-directed instruction.
Ørbæk and Engelsrud (2021)	Creative-dance pedagogy evolving from set-movement instruction toward expressive exploration and movement-emotion-language connections	Cognitive outcomes; Implementation outcomes	The pedagogy evolved toward explicit connections among movements, emotions, and language for teaching and learning. The study documented an evolution from instructing set movements and facilitating expressive exploration toward a more relational pedagogy attentive to intercorporeal and interaffective dynamics.
Park (2017)	Esthetic-experience-oriented dance education emphasizing imagination, expression, creation, appreciation, varied stimuli, autonomous methods, and group activity; the study derived the Ae-SD strategy	Psychosocial outcomes; Implementation outcomes	Group activity and communication were described as important routes through which learners sensed, shared, and interpreted movement experiences together. The study derived the Ae-SD strategy, organizing esthetic-experience pedagogy around esthetic focus, student-centredness, and diversity-centered teaching.
Powell (2025)	Feminist and democratic dance-technique pedagogy using open questions, discussion, shared decision-making, collaboration, and differentiation to challenge authoritarian teaching	Cognitive outcomes; Psychosocial outcomes; Implementation outcomes	The reflective account emphasized that active participation and questioning can promote critical thinking, reflection, and deeper learning. The class was described as potentially becoming a collaborative learning environment in which reciprocity and collective growth are strengthened. The article emphasized that many students expect to be shown exactly what to do and may resist democratic participation because prior training has normalized.
Rachel Rimmer (2017)	Enquiry-based, constructivist dance-technique pedagogy using partner problem-solving, phrase creation, improvisation, discussion, and journal reflection to support active learner agency	Cognitive outcomes; Implementation outcomes; Participation-related outcomes	The enquiry-based approach was designed to and was discussed as helping students become more reflective and more active agents in learning dance technique. The study highlighted how doxic expectations of dance technique as rigid, precise, and teacher-led complicated attempts to implement enquiry-based pedagogy.
Rimmer (2017)	Dialogic, somatic-informed teaching that returned questions to students, foregrounded first-person reflection, used exploratory tasks, peer observation, peer feedback, improvisation, and collaborative discussion to challenge hierarchical technique teaching.	Cognitive outcomes; Psychosocial outcomes; Implementation outcomes	Dialog allowed students to reflect on their understanding of technical concepts individually and with others, helping meaning making around technique. Peer tasks facilitated further dialog and awareness, but the article also notes tensions around peer judgment, imitation, and possible danger in dialogic interaction.
Rimmer-Piekarczyk (2018)	The study analyzed student learning experiences across a continuum from teacher-centered reproductive technique work toward more student-centered explorative, improvisational, and self-regulated learning practices.	Psychosocial outcomes; Implementation outcomes	Student-centered experiences involved greater activity, choice, and altered power relations, though not without tension. The article highlights nuanced tensions, especially early in the program, where some students still sought more teacher-centered clarity and correction.
Rothmund (2023)	The study mapped naturally occurring instruction, questioning, feedback, and focus-of-attention practices to characterize how teaching information was delivered across class types.	Implementation outcomes	Improvisation showed the highest positive–negative ratio, whereas one ballet class had a ratio below 1, indicating substantial between-class variation. Direct feedback behaviors were highly prevalent and clustered especially after exercises, indicating a feedback-heavy teaching ecology. Open questions were relatively rare outside improvisation, suggesting limited but class-specific support for reflective self-regulation.
Vincent et al. (2021)	The study compared teacher-reported delivery of certificated dance curricula, highlighting a more integrated, resource-based and partially student-centered GCSE approach versus more compartmentalized National 5 delivery with variable discovery learning	Implementation outcomes; Psychosocial outcomes	GCSE teachers described using the anthology of professional works to link analysis, performance, and creation more holistically, aligning more closely with the Midway Model than the Scottish context. Teachers in both contexts described certificated dance as fostering communication, confidence, relationship-building, self-reflection, and other transferable capabilities beyond technical performance. English teachers reported substantial AQA.
Wilson (2009)	Somatic experiential-anatomy pedagogy that presented anatomical and kinesiological concepts and then asked dancers to explore, embody, journal, discuss, and apply them in movement and technique contexts	Cognitive outcomes	Across the cases, the data supported the theme that embodied experience developed into knowledge, suggesting that somatic movement exploration helped students make meaning from anatomical information. The synthesis indicated that embodied anatomical learning unfolded over time, rather than through immediate one-off acquisition.

## 4 Discussion

Across the included studies, the most consistent signal was not a simple superiority of one pedagogical pole over the other, but a recurring advantage of pedagogies that gave learners an active role in making sense of movement through reflection, dialog, collaboration, observation, and choice. Importantly, this pattern was supported most strongly by qualitative, mixed-methods, and implementation-oriented evidence concerning cognitive, self-related, psychosocial, and pedagogical-process outcomes. By contrast, the comparative quantitative evidence bearing directly on relative effectiveness was narrower and methodologically weaker, especially for technical/performance outcomes, where findings were mixed rather than strongly directional. The included studies also showed that the field rarely studies pure pedagogical models. Instead, most studies described hybrid instructional ecologies in which demonstration, correction, questioning, exploratory tasks, and peer exchange were combined in different proportions.

The more useful interpretive question, however, is not simply whether learner-centered pedagogy is better than teacher-directed pedagogy, but when, for whom, and along which dimensions greater learner participation supports different outcomes. The review suggests that this continuum is multidimensional, involving at least the distribution of decision rights, the control and timing of feedback, and the openness of the task with respect to reproduction versus interpretation. Framed in this way, the evidence points less to a single optimal pedagogy than to context-sensitive combinations whose value depends on learner experience, genre conventions, task demands, and educational aims.

### 4.1 Learner-centered and teacher-directed pedagogy are better understood as a multidimensional, context-dependent continuum

One of the clearest interpretive findings of the review is that the opposition between learner-centered and teacher-directed pedagogy is analytically useful but pedagogically incomplete. What varies is not only how much teachers direct, but also who controls decisions, how feedback is mediated, and how open the task is to interpretation. Several included studies described dance teaching as a continuum rather than a binary choice. In Korean dance education, a practice-based framework explicitly organized pedagogy across transmission, facilitation, and co-construction rather than treating these as mutually exclusive modes (Jung et al., 2026). In ballet, direct and indirect methods were both presented as necessary components of whole-person dance education, with hands-on correction, demonstration, and repetition coexisting with imagination, recommendation, reflection, and self-understanding (Choi and Kim, 2015). Student accounts of contemporary technique learning similarly suggested that experiences shifted across a continuum from reproductive, teacher-centered work toward more explorative and student-centered learning, with value and difficulty attached to both ends of that continuum (Rothmund, 2023).

This continuum perspective is also important because it helps explain why apparently learner-centered pedagogies often retained substantial teacher structuring, and why apparently teacher-directed pedagogies were more effective when they included adaptive feedback and opportunities for learner interpretation. Assessment-oriented dance teaching in upper secondary education still relied on demonstration and teacher judgment, but it became more educative when it created space for student independence, self-monitoring, and embodied understanding (Andersson, 2018). Observational work in contemporary dance classes showed that actual studio teaching remained dominated by direct feedback and relatively limited open questioning, even in settings that valued learner development (Soerel et al., 2023). Conversely, the prospective observational study of Cecchetti ballet classes suggested that teacher quality mattered, not simply teacher directiveness: clearer demonstration, task-relevant cueing, and congruent feedback were associated with better performance gains (Lee, 2009). Taken together, these studies suggest that the central pedagogical issue is less whether teachers direct learning and more how they direct it, when they release responsibility, and how they support learners to interpret rather than merely reproduce movement material. From this pattern, some provisional propositions follow. Initial modeling and explicit cueing may be especially important for novices, safety-relevant content, or high-precision technical goals. Once a basic task frame is established, exploratory rehearsal, questioning, and learner interpretation may support deeper understanding, self-monitoring, and transfer. Moreover, feedback may be most educationally productive when learners first attempt movement, then analyze it, then revise and re-perform, rather than receiving correction alone. Finally, as learner competence increases, decision-making can be progressively redistributed toward learners without abandoning teacher guidance.

### 4.2 Dance learning and performance outcomes may favor active sense-making, but not uniform superiority over structured direct teaching

Across the studies that addressed dance learning and performance directly, learner participation in analysis, exploration, and embodied meaning-making was repeatedly described as beneficial. Somatic and dialogic approaches in ballet and contemporary dance were associated with deeper body awareness, self-correction, and more adaptive engagement with technique rather than reliance on external correction alone (Berg, 2017; Rimmer-Piekarczyk, 2018). Care-oriented and transformation-focused technique pedagogy similarly positioned students as active participants in the learning process and linked that position to both bodily and personal development (Dryburgh, 2019). In creative and expressive dance contexts, students were reported to learn through experimentation, collaboration, and attempts to connect movement with feeling, language, or intention rather than through imitation alone (Engdahl et al., 2024; Larsson et al., 2025; Ørbæk and Engelsrud, 2021). Related patterns were also visible in study-abroad, somatic-anatomy, and esthetic-experience work, where technique and learning were presented as something constructed through embodied inquiry and reflection rather than delivered as fixed content (Mabingo, 2019; Wilson, 2009).

At the same time, the comparative evidence does not justify a strong claim that learner-centered pedagogies are uniformly more effective than more directive teaching for technical achievement. In the clearest classroom comparison, both the student-centered peer-feedback condition and the direct-instruction condition improved significantly across most rubric criteria, but the learner-centered condition did not outperform direct instruction after adjustment (Brown, 2023). By contrast, online dance-analysis teaching was associated with a more favorable distribution of assessment results than traditional teaching in one non-randomized comparison (Ma, 2024), and digitally mediated feedback systems were described positively in another quasi-experimental study, although that evidence had substantial design limitations (Chen, 2025). The observational comparison by Lee (2009) further complicates any anti-directive interpretation because it suggests that highly structured teaching can support performance when the instruction is clear, specific, and feedback-rich. A more defensible interpretation is therefore that performance learning benefits when teacher guidance is combined with opportunities for analysis, reflection, and learner adjustment, rather than when one pedagogy simply replaces the other.

The evidence for motor and physical outcomes was especially sparse and should be interpreted cautiously. Only a small number of studies discussed outcomes such as kinesthetic awareness, coordination, balance, alignment, or pain management, and most of these accounts were qualitative or descriptive rather than comparative (Berg, 2017; Guo and Thiagarajan, 2025; Kim and Yi, 2022; Wilson, 2009). These findings support the plausibility of somatic, exploratory, and aestheticaly oriented pedagogies for body awareness and movement organization, but they are not yet sufficient to establish comparative effectiveness. This domain remains a meaningful gap, especially given how central bodily regulation and physical adaptation are to dance pedagogy in practice.

### 4.3 Cognitive, self-related, and motivational-affective outcomes form the strongest and most coherent pattern in the evidence base

The review found its strongest and most internally consistent pattern in cognitive and self-related outcomes. Across creative, somatic, dialogic, portfolio-based, and critically oriented pedagogies, learners were repeatedly described as developing stronger reflective capacity, conceptual understanding, self-observation, and ownership of learning (Guo and Thiagarajan, 2025; Nam, 2010; Rimmer, 2017; Rimmer-Piekarczyk, 2018; Wilson, 2009). These outcomes can also be interpreted through frameworks of self-regulated learning, metacognition, and reflective practice. Specifically, the reported benefits in self-observation, ownership, and adaptive adjustment map closely onto self-regulated learning processes of planning, monitoring, evaluating, and modifying performance. They also suggest metacognitive development insofar as learners become more aware of what they are doing, why they are doing it, and how to change it. From a reflective-practice perspective, dialogic, somatic, and portfolio-based pedagogies appear to deepen both reflection-in-action and reflection-on-action within dance learning.

Inquiry-oriented studies in technique and physical education also suggested that open tasks and exploratory assignments helped learners attend to meaning, choice, and movement possibilities rather than only to externally prescribed forms (Larsson et al., 2025; Mattsson and Larsson, 2021; Ørbæk and Engelsrud, 2021). In higher education settings, digital and narrative pedagogies similarly made learning processes more visible, whether through video-supported reflection, anonymous peer assessment, or frameworks for identifying otherwise invisible graduate attributes (Hsia and Sung, 2020; Huddy, 2017; Longley and Kensington-Miller, 2020). The cumulative implication is that learner-centered pedagogy may be especially valuable when the educational aim includes understanding, interpretation, self-regulation, and transfer, not only task execution.

The comparative studies reinforce this interpretation, although again with important nuance. Problem-solving teaching produced higher cognitive participation than command teaching in one mixed-methods comparison, and many students preferred it because it allowed freedom, creativity, and discovery (Cuellar-Moreno and Caballero-Juliá, 2019). Mobile peer assessment in a flipped class was associated with stronger intrinsic motivation, greater reflection, and more active observation than instructor feedback alone, while self-efficacy and motivation were positively related (Hsia and Sung, 2020). At the same time, motivational and confidence effects were not uniformly favorable. Brown (2023) found reduced comfort with peer feedback in the student-centered condition, García-Dantas and Quested (2015) did not identify significant between-group self-efficacy differences across feedback conditions, and Rothmund (2023) showed that some students valued student-centered learning more over time than at the beginning of the course. These findings suggest that learner-centered pedagogies may strengthen agency and reflection most reliably when they are scaffolded developmentally and socially, especially for adolescents, novice learners, or students accustomed to more hierarchical dance cultures.

Qualitative self-study and practitioner-focused work adds an important interpretive layer to these findings. Several studies described shifts away from coercive, rigid, or apprenticeship-like teaching toward more enjoyable, relational, and learner-responsive pedagogies, and linked those shifts to better body awareness, stronger identification with dance, and more positive attitudes toward learning (Lim and Lee, 2020; Mi-Hee, 2016). Studies of esthetic experience, critical dance pedagogy, and feminist pedagogy likewise framed self-related outcomes not simply as confidence gains, but as changes in voice, autonomy, identity, and the capacity to locate one’s own experience within a broader social and artistic context (Marani et al., 2022; McCarthy-Brown, 2021; Park, 2017; Powell, 2025). This broader interpretation is important because it suggests that the value of learner-centered pedagogy in dance is not restricted to motivational enhancement. It may also reshape how learners understand themselves as moving, interpreting, and socially situated subjects.

### 4.4 Psychosocial and sociocultural outcomes become most visible when pedagogy is collaborative, dialogic, and inclusion-oriented

Psychosocial outcomes were among the clearest strengths of the learner-centered literature. Across creative dance, portfolio-based work, digitally mediated collaboration, and dialogic technique pedagogy, students were reported to benefit from cooperation, peer learning, mutual respect, and shared responsibility for meaning-making (Huddy, 2017; Nam, 2010; Rimmer-Piekarczyk, 2018; Rothmund, 2023). In these studies, psychosocial development was not an incidental by-product, but a direct consequence of pedagogies that required learners to observe one another, negotiate ideas, give feedback, and take responsibility within a group. Even where the primary outcome of interest was not social, the pedagogical mechanisms that supported learning were often interpersonal.

The psychosocial dimension also broadened substantially in studies that foregrounded inclusion, gender, race, and cultural encounter. Study-abroad dance education in Uganda was described as producing intercultural learning and relational awareness while also surfacing ethical tensions around representation and appropriation (Mabingo, 2019). Critical and feminist dance pedagogies extended psychosocial outcomes toward dialog about power, identity, reciprocity, and collaboration rather than limiting them to classroom cohesion (McCarthy-Brown, 2021; Powell, 2025). Work in physical education and school settings likewise suggested that expressive or body-percussion pedagogies can challenge gender stereotypes, widen access, and create alternative conditions for participation, although such outcomes remain under-studied longitudinally (Feltham and Ryan, 2022; de Garcías Ves and Beltran-Garrido, 2024; Jo and Lee, 2022; Mattsson and Larsson, 2021). Vincent et al. (2021) further showed that institutional curriculum structures can either support or narrow these broader educational aims. These findings point to some possible priorities for future research. Studies should test which pedagogical components (such as learner choice, collaborative choreography, dialogic reflection, inclusive language, culturally responsive repertoire, or varied role structures) most consistently support inclusion across gender, race, and cultural background. Moreover, technique-class research should examine how authority, correction, touch, demonstration, and evaluative power are negotiated, redistributed, or resisted across different training cultures. Finally, longitudinal and mixed-methods designs are needed to evaluate whether these pedagogies shape belonging, identity, retention, and long-term participation beyond single courses or short interventions.

### 4.5 Implementation findings show that pedagogical change depends on assessment cultures, teacher learning, and institutional support

Implementation outcomes were the most frequently reported in the review, and this is a crucial finding in itself. The literature repeatedly indicated that learner-centered pedagogy is demanding to enact, not simply desirable to endorse. In school and higher education settings, teachers described tensions involving time, curricular pressure, assessment systems, infrastructure, learner readiness, and uneven professional support (Andersson, 2018; Jo and Lee, 2022; Vincent et al., 2021). Contemporary studio observation further suggested that actual instruction remains heavily weighted toward directive feedback, even where broader pedagogical discourse values reflection and autonomy (Soerel et al., 2023). Digitally enhanced approaches offered one possible route to support observation, rehearsal, and feedback, but their benefits were still dependent on design quality, platform use, and pedagogical integration rather than technology alone (Chen, 2025; Huddy, 2017; Hsia and Sung, 2020).

Several studies also suggest that implementing learner-centered dance pedagogy requires substantial teacher learning and identity work. Self-study and autoethnographic research showed that movement away from coercive or transmission-heavy teaching was tied to teachers’ own reflective change, not merely to a new classroom technique (Lim and Lee, 2020; Mi-Hee, 2016). Practice-based and feminist accounts likewise implied that learner-centered teaching depends on clear values, explicit framing, and the ability to hold structure and openness together rather than simply reducing authority (Jung et al., 2026; Powell, 2025). Brown (2023) is instructive here, because the mixed findings around peer feedback suggest that simply inserting a student-centered practice into a class is insufficient if the social and evaluative conditions needed for productive peer exchange are not deliberately scaffolded. The implementation record therefore supports a cautious but practical conclusion: pedagogical change in dance is most likely to succeed when it is treated as a curriculum, assessment, and teacher-development issue rather than as a single classroom technique.

### 4.6 Limitations of the included evidence, gaps in risk of bias, and future research

The conclusions that can be drawn from this review are constrained by the character of the evidence base. Most included studies were qualitative, and much of the literature came from higher education contexts in Europe and Asia rather than from school, community, professional, or long-term training settings. The qualitative evidence was often informative and contextually rich, but it was usually appraised as moderate rather than unequivocally robust, chiefly because reflexivity, philosophical positioning, and ethics reporting were inconsistent. The quantitative and comparative evidence was much thinner and methodologically weaker. Among the quasi-experimental studies contributing to the synthesis, most were judged to be at high risk of bias because of non-randomized allocation, weak or absent control conditions, limited pre-intervention and post-intervention measurement, incomplete handling of follow-up, and insufficiently detailed statistical reporting. Accordingly, the practical implications should be read as provisional and context-sensitive rather than as evidence that learner-centered pedagogy is generally superior to more directive teaching. The strongest defensible implication is that certain learner-centered components appear relevant for some outcome domains and contexts, especially when integrated with appropriate teacher guidance, but the present evidence does not justify broad claims of pedagogical superiority.

The randomized studies were more promising but still raised some concerns, especially around randomization reporting, outcome measurement, and the absence of pre-specified analysis plans. A small number of eligible reports also had reporting limitations severe enough to reduce confidence further, including abstract-only evidence and studies with under-specified samples or analyses. In addition, because the review necessarily relied on narrative synthesis across heterogeneous pedagogies, settings, and outcome constructs, the present discussion should be interpreted as structured evidence mapping rather than as a substitute for pooled effect estimation. Direct dance learning/performance outcomes as the primary domain, and more tentatively cognitive/self-regulatory outcomes where repeated patterns were observed across studies. Other outcome families, especially motor/physical, participation-related, and broader psychosocial outcomes, are better interpreted at present as mapped areas of emerging evidence rather than as domains supporting firm comparative inference.

The evidence gap map sharpens these limitations by showing where knowledge remains thin. Motor and physical outcomes and participation-related outcomes were notably sparse, and acute or single-session studies contributed only a small minority of the mapped evidence. The field also lacks a stable core set of outcomes, common reporting standards for pedagogical dose and fidelity, and comparative designs that can disentangle pedagogy from genre, teacher expertise, learner level, and curriculum context. Future studies would therefore benefit from multi-site and adequately powered controlled designs, clearer descriptions of comparison conditions, longer follow-up periods, and combined outcome frameworks that include technique learning, bodily adaptation, motivation, self-regulation, social climate, inclusion, and retention. There is also a strong case for more implementation-focused effectiveness research: studies should not only ask whether a pedagogy works, but for whom, in which genres and institutions, through which mechanisms, and under what conditions teachers can sustain it. Finally, given the recurring continuum pattern in the present review, future research should move beyond crude learner-centered versus teacher-centered labels and instead test specific pedagogical components, sequences, and combinations.

### 4.7 Practical applications and next steps

For practice, the findings support neither a return to purely authoritarian dance teaching nor an unstructured celebration of learner freedom. The more useful implication is that teachers should design learning environments in which direct instruction, demonstration, and correction are used strategically, but are complemented by opportunities for questioning, reflection, peer dialog, exploratory problem solving, and learner choice. These implications should, however, be bounded by context. In school and physical-education contexts, especially where the aims include participation, expression, confidence, and creativity, exploratory and dialogic pedagogies may be especially valuable. In conservatory and technique-intensive studio contexts, particularly for novice learners or precision-demanding genre conventions, explicit modeling, correction, and guided repetition may remain foundational, with learner-centered elements introduced through reflection, interpretation, and progressively increased responsibility. In higher-education and contemporary/choreographic contexts, where independent meaning-making and self-regulation are often explicit aims, peer dialog, self-evaluation, and exploratory task design may warrant a more central role. That interpretation is consistent across ballet, contemporary, creative, and digitally mediated settings, even though the exact balance should vary by learner experience, genre conventions, and task demands (Berg, 2017; Brown, 2023; Choi and Kim, 2015; Hsia and Sung, 2020; Rimmer-Piekarczyk, 2018). In practical terms, this means sequencing rather than polarizing pedagogies: technical material may first need to be modeled clearly, then explored, discussed, adapted, and re-performed so that learners can develop both accuracy and ownership.

The findings also suggest several next steps for curriculum and teacher education. Peer feedback and collaborative work appear promising, but they should be explicitly taught, socially scaffolded, and aligned with psychologically safe assessment cultures rather than assumed to function automatically (Andersson, 2018; Brown, 2023). Digital tools can enrich rehearsal, observation, and reflection, but they need to serve pedagogical purposes rather than displace them (Chen, 2025; Huddy, 2017). Teacher education programs may also benefit from stronger preparation in reflective questioning, feedback design, dialogic facilitation, somatic awareness, and the management of power relations in the studio, especially where dance cultures have historically normalized narrow authority structures (Jung et al., 2026; Lim and Lee, 2020; Powell, 2025; Mi-Hee, 2016). At program level, a productive next step would be to align pedagogy, assessment, and curriculum documents so that valued outcomes such as agency, reflection, intercultural understanding, and collaborative learning are assessed and supported as deliberately as technical execution.

## 5 Conclusion

This review maps an emerging but uneven evidence base on learner-centered and teacher-directed dance pedagogy. The strongest pattern is that learner participation, reflection, dialog, and collaborative meaning-making are repeatedly associated with cognitive, self-related, psychosocial, and implementation benefits, while direct evidence of superior technical effectiveness over structured teacher guidance remains mixed. The review therefore supports a scoping-style conclusion rather than a definitive causal one. The literature points toward the value of well-scaffolded hybrid pedagogies that balance clear instruction with growing learner agency, but it also shows that the field still needs stronger comparative, longitudinal, and implementation-sensitive research before firmer effectiveness claims can be made. For now, the most defensible conclusion is that how dance is taught matters profoundly, and that the most educationally rich approaches are those that treat technique, interpretation, reflection, and relationship as mutually reinforcing rather than pedagogically separate. In lesson-design terms, this means integrating connected principles, namely clear modeling of task demands, guided exploration and interpretive choice, dialogic/self/peer-informed feedback, and opportunities to revise and re-perform with increasing learner responsibility.

## Data Availability

The original contributions presented in the study are included in the article/supplementary material, further inquiries can be directed to the corresponding authors.
